# Ab Initio Values of the Thermophysical Properties of Helium as Standards

**DOI:** 10.6028/jres.105.054

**Published:** 2000-10-01

**Authors:** John J. Hurly, Michael R. Moldover

**Affiliations:** National Institute of Standards and Technology, Gaithersburg, MD 20899-8360

**Keywords:** diffusion coefficient, helium, intermolecular potential, second virial, thermal conductivity, thermal diffusion factor, thermophysical standards, transport properties, viscosity

## Abstract

Recent quantum mechanical calculations of the interaction energy of pairs of helium atoms are accurate and some include reliable estimates of their uncertainty. We combined these *ab initio* results with earlier published results to obtain a helium-helium interatomic potential that includes relativistic retardation effects over all ranges of interaction. From this potential, we calculated the thermophysical properties of helium, i.e., the second virial coefficients, the dilute-gas viscosities, and the dilute-gas thermal conductivities of ^3^He, ^4^He, and their equimolar mixture from 1 K to 10^4^ K. We also calculated the diffusion and thermal diffusion coefficients of mixtures of ^3^He and ^4^He. For the pure fluids, the uncertainties of the calculated values are dominated by the uncertainties of the potential; for the mixtures, the uncertainties of the transport properties also include contributions from approximations in the transport theory. In all cases, the uncertainties are smaller than the corresponding experimental uncertainties; therefore, we recommend the *ab initio* results be used as standards for calibrating instruments relying on these thermophysical properties. We present the calculated thermophysical properties in easy-to-use tabular form.

## 1. Introduction

**Fig. 1:**
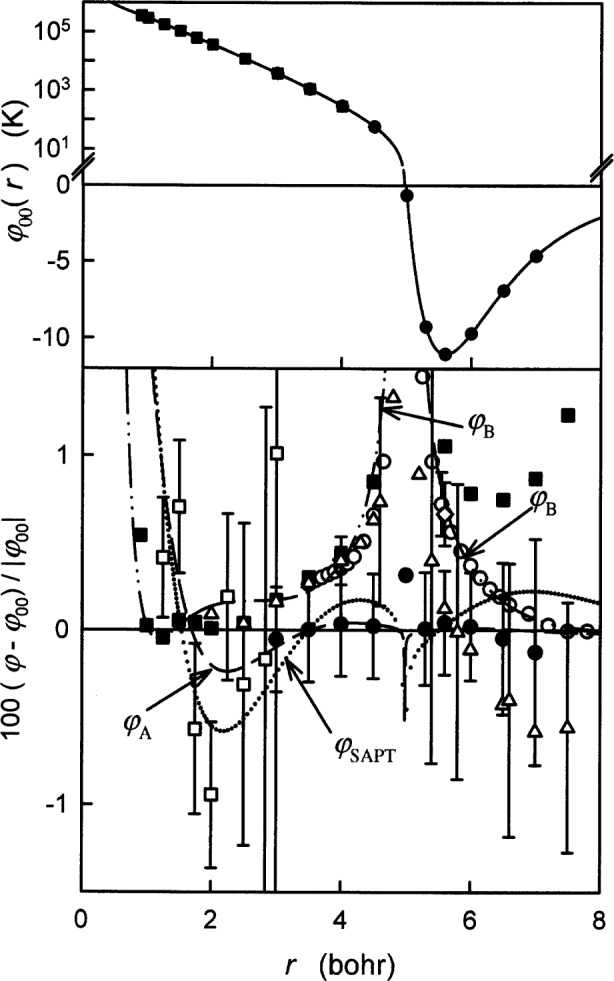
Top: the helium-helium pair potential *φ*_00_(*r*). Note: a logarithmic scale is used for positive values of *φ*_00_(*r*) and a linear scale is used for negative values of *φ*_00_(*r*). Bottom: the uncertainties of the *ab initio* results and their fractional deviations from *φ*_00_(*r*). Also shown are the fractional deviations of the considered potentials fit to the various *ab initio* values. The fractional deviations diverge near *r* = 5.0 bohr where *φ*_00_(*r*) passes through zero. Key: (— —) *φ*_A_; (• • • • • •) *φ*_SAPT_; (— • •) *φ*_B_; □ Ceperley and Partridge [15]; ■ Komasa [24]; ● Korona et al. [18]; ○ van Mourik and Dunning [21]; ◇ van de Bovenkamp and van Duijneveldt [20]; △ Gdanitz [19].

Today, the most accurate values of the thermophysical properties of helium at low densities can be obtained from two, very lengthy, calculations. The first calculation uses quantum mechanics and the fundamental constants to obtain, *ab initio*, a potential energy *φ*(*r*) for the helium-helium (He_2_) interaction at discrete values of the interatomic separation *r* and also limiting forms of *φ*(*r*) at large *r* (see Fig. 1). The second calculation uses standard formulae from quantum-statistical mechanics and the kinetic theory of gases to obtain the thermophysical properties of low-density helium from *φ*(*r*). Here, we report the results of the second calculation spanning the temperature range 1 K to 10^4^ K for the second virial coefficient *B*(*T*), the viscosity *η*(*T*), the thermal conductivity *λ*(*T*), the mass diffusion coefficient *D*(*T*), and the thermal diffusion factor *α*_T_(*T*) for ^3^He, ^4^He, and their equimolar mixture. Our results, together with estimates of their uncertainties, are presented in easy-to-use tabular form in Appendix A. For the pure fluids, the statistical-mechanics calculations make negligible contributions to the uncertainties of the tabulated properties; therefore, we estimated the uncertainties of the results by varying *φ*(*r*) within its uncertainty and examining the consequences. For the equimolar mixture, the results from different orders of approximation in the transport theory are compared to estimate their contribution to the uncertainties.

The present results can be applied to many problems in metrology; here we mention a few. Low-density helium is used in primary, constant-volume, gas thermometry [1]; primary, dielectric-constant gas thermometry [2]; and in interpolating gas thermometry (required by ITS-90 in the temperature range 3 K to 24.6 K) [3]. These applications require the extrapolation of measurements to zero pressure. If the present values of *B*(*T*) are used for such extrapolations, the results may be more accurate and the probability of detecting systematic errors in the measurements will be increased. Low-density helium can be used to calibrate acoustic resonators for acoustic thermometry and for measuring the speed of sound in diverse gases. Spherical acoustic resonators [4] may be calibrated using the present values of *λ*(*T*), *B*(*T*), and temperature derivatives d*B*/d*T* and d^2^*B*/d*T*^2^. The same properties together with *η*(*T*) may be used to calibrate cylindrical acoustic resonators [5]. Other instruments that might be calibrated with the help of the present results include the vibrating wire viscometer [6], the Greenspan acoustic viscometer [7], and the Burnett apparatus [8] for making very accurate measurements of the equation of state of moderately dense fluids.

The present work contrasts with a long tradition of using semi-empirical models for *φ*(*r*) to correlate the thermophysical property data for helium and the other monatomic gases [9, 10, 11]. These semi-empirical models combined limited *ab initio* results with critically evaluated and judiciously selected experimental data to determine the function *φ*(*r*) that correlates as much data as possible. In this work, we did not consider experimental results until all of the calculations were completed as in [12, 13]. The *ab initio* results were then compared to the sets of data that others had selected as inputs to semi-empirical models. In every case that we examined, the *ab initio* values of the thermophysical properties agreed with the data within plausible estimates of their combined uncertainties.

This manuscript is organized as follows: Sec. 2 reviews the *ab initio* results for *φ*(*r*) and our analytic representation of them. Section 3 outlines the steps in calculating the thermophysical properties of helium from *φ*(*r*). Each step includes a description of the precautions that were taken to insure that imperfections of the numerical methods did not adversely affect the results. Section 4 estimates the uncertainty of the *ab initio* helium pair potential and how it propagates into the uncertainties of the calculated properties. Section 5 describes the tabulated results and methods for their use. Section 6 compares the calculated properties with selected measurements. Section 7 summarizes the present results and the prospects for future refinements.

## 2. *Ab Initio* Values for the He_2_ Potential Energy Functions *φ*(*r*)

Table 1 lists recent *ab initio* values of *φ*(*r*) at selected values of *r* (3.0 bohr, 4.0 bohr, and 5.6 bohr, where 1 bohr = 0.052917721 nm) and, where available, the uncertainties estimated by the original authors. As is conventional in this field, the potential energy is divided by *k*_B_ K and thus has the unit K (*k*_B_ is the Boltzmann constant [14] and K is the unit symbol for the kelvin). The various calculations almost, but not quite, agree within their uncertainties. The discrepancies near 4.0 bohr are particularly significant in determining the uncertainties of thermophysical properties of helium near ambient temperatures. A detailed evaluation of each calculation in Table 1 is beyond the scope of this paper. Here, we mention the observations that guided our selection among the sources cited in Table 1 to obtain *φ*_00_(*r*), the function that we used to calculate the thermophysical properties of helium.

### 2.1 Long-Ranges: *r* ≳ 8 bohr

The asymptotic long-range attractive behavior of our preferred potential *φ*_00_(*r*) is represented by the two-body dispersion coefficients *C_n_* (*n* = 6, 8, …) in the multipole expansion. These coefficients have been calculated, *ab initio*, by two independent groups [22, 23] using a sum-over-states formalism with explicitly electron-correlated wave functions to describe the states. The independent calculations [22, 23] differed by less than 1 in the fourth digit. This small difference makes a negligible contribution to the uncertainties of the thermophysical properties calculated from *φ*_00_(*r*).

### 2.2 Short-Ranges: *r* ≲ 3 bohr

Ceperley and Partridge [15] obtained values of *φ*(*r*) at small *r* using a quantum Monte Carlo (QMC) method. The QMC method is exact insofar as it requires no mathematical or physical approximations beyond those in the Schrödinger equation and the method yields estimates of the uncertainties of *φ*(*r*). Komasa [24] used a variational method to obtain rigorous upper bounds to *φ*(*r*) in the range 0.01 bohr ≤ *r* ≤ 15 bohr. At some values of *r*, the variational values of *φ*(*r*) are less than the QMC values; however the differences between the values are usually within twice the QMC uncertainties. Thus, we used the variational values to determine *φ*_00_(*r*) and we have evidence that the QMC uncertainties are reasonable. At smaller values of *r* the variational and QMC results are inconsistent. For example, at *r* = 1 bohr (not plotted), Komasa reports *φ*(1 bohr) = (286.44 ± 0.03) × 10^3^ K, and Ceperley and Partridge report *φ*(1 bohr) = (291.9 ± 0.6) × 10^3^ K. We are unable to resolve this inconsistency; however, the inconsistency does not affect the thermophysical properties in the temperature range 1 K to 10^4^ K.

Komasa provides two values for the well depth at 5.6 bohr, *ε*/*k*_B_ = −10.947 K using a 1200-term basis set and *ε*/*k*_B_ = −10.978 K using a 2048 term basis set. The second value is 0.3 % lower. Komasa’s calculations at other values of *r* used the 1200-term basis set. We speculate that comparable reductions in *φ*(*r*) would occur if Komasa’s variational calculation were repeated with the larger basis set at all values of *r*.

### 2.3 Intermediate Ranges: 3 ≳ *r* ≳ 8 bohr

At intermediate ranges, we considered the seven relevant publications cited in Table 1. Anderson et al. [16] report exact QMC results that have relatively large uncertainties. Klopper and Noga [17] used an explicitly correlated coupled cluster [CCSD(T)] method that resulted in the limiting value for the well depth of *ε*/*k*_B_ = −10.68 K at 5.6 bohr. Then, they estimated the effects of quadruple substitutions to be −0.32 K at 5.6 bohr (and −1.9 K at 4.0 bohr) by comparing their results to the full configuration interaction (FCI) calculation of van Mourik and van Lenthe [25]. This extrapolation to a complete basis set resulted in *ε*/*k*_B_ = −(11.0 ± 0.03) K, which agrees with the QMC results of Anderson [16].

Korona et al. [18] used symmetry-adapted perturbation theory (SAPT) to calculate values for *φ*(*r*) with uncertainties that they estimated to be the larger of 0.3 % or 0.03 K in the range 3 bohr ≤ *r* ≤ 7 bohr. The SAPT well-depth is *ε*/*k*_B_ = −(11.06 ± 0.03) K, the lowest of all *ab initio* results; however, it also agrees with the QMC result [16] within the latter’s uncertainty.

While this project was in progress, two groups extended the CCSD(T) calculations of Klopper and Noga [17]. These groups (de Bovenkamp and Duijneveldt [20]; and van Mourik and Dunning [21]) used different techniques to extrapolate the results of Klopper and Noga [17] to an infinite basis set. Gdanitz [19] also published calculations labeled r12-MRACPF in which he extrapolated his results to an infinite basis set by yet another method. These three recent publications and the variational results of Komasa [24] indicate that the SAPT [18] results in the region around *r* = 4.0 bohr are too attractive by approximately 0.05 K (Fig. 1, lower panel). Nevertheless, we used the SAPT intermediate-range results in determining the potential *φ*_00_(*r*) and we used the differences between the SAPT and the other results to determine alternative potentials that were used to estimate the uncertainties of the thermophysical properties. Our decisions are based on three observations. First, we recalled that Komasa’s [24] variational result at 5.6 bohr decreased 0.3 % upon increasing the basis set from 1200 terms to 2048 terms. If Komasa’s result at 4.0 bohr (292.784 K) were decreased by 0.3 %, it would be 291.906 K, in agreement with the SAPT value of 291.64 K. Gdanitz suggested that the decrease at 4.0 bohr might be less than 0.3 % because the variation method is more accurate at smaller separations [26]. Second, we noted that the two extensions of Klopper and Noga’ work [17] are not independent. The two decompose *φ*(*r*) in several components, the largest of which were calculated best by Klopper and Noga. Thus, the uncertainties of these results may be dominated by those of Klopper and Noga. (van Mourik and Dunning [21] state, “It is likely that the corrected curve is the most accurate available to date for He_2_ interactions”. In effect, they asserted that Klopper and Noga’s interaction energies are more accurate than their own complete basis set extrapolated energies.) Third, Bukowski et al. [27] argue that their own Gaussian-type geminals (GTG) computation bounds the larger components of Klopper and Noga’s CCSD(T) computations and they suggest that Klopper and Noga’s results may be too high by approximately 0.3 K at 4 bohr and by approximately 0.04 K at 5.6 bohr. If Bukowski et al.’s suggestion is correct and if one decreases the CCSD(T) values of *φ*(*r*) accordingly, then they all would agree with the SAPT results. Ultimately, additional calculations will resolve these issues.

### 2.4 Algebraic Representations of *ab initio* Values of *φ*(*r*)

We calculated the thermophysical properties of helium six times, each using a different function to represent *ab initio* values of *φ*(*r*). We fitted two of these six functions, *φ*00 and *φ*_B_, to our own selections among the published *ab initio* values. The third function, *φ*_SAPT_, had already been fitted by others to *ab initio* results and used to calculate thermophysical properties. [18] We fitted the fourth, *φ*_A_, to the same *ab initio* results used to obtain *φ*_SAPT_; however, we added one additional fitting parameter. Thus, differences between the thermophysical properties computed from *φ*_SAPT_ and *φ*_A_ provide one indication of the sensitivity of the properties to the algebraic representation of the *ab initio* “data”. The last two functions are denoted, $\varphi_{A}^{-}$, and $\varphi_{A}^{+}$. To obtain $\varphi_{A}^{-}$, we decreased the *ab initio* short-range results [15] by their claimed uncertainties and decreased the intermediate-range SAPT results by 0.1 % and re-fitted them. Then, we increased the *ab initio* results by their claimed uncertainties and the SAPT results by 0.1 % and fitted them to obtain $\varphi_{A}^{+}$. The differences between the thermophysical properties calculated using *φ*_A_, $\varphi_{A}^{-}$, $\varphi_{A}^{+}$ and *φ*_SAPT_ are analogous to the uncertainties of measured values of thermophysical properties conducted in a single laboratory and analyzed using different methods. In the present case, the differences between the thermophysical properties calculated from *φ*_A_, $\varphi_{A}^{-}$, $\varphi_{A}^{+}$, and *φ*_SAPT_ are much smaller than the differences between those calculated from *φ*_00_, *φ*_A_, and *φ*_B_.

#### 2.4.1 *φ*_00_

We used *φ*_00_ to calculate the thermophysical properties tabulated in Appendix A. In our judgement, *φ*_00_ is the best representation of the *ab initio* results available at the time of this writing. The subscript “00” identifies *φ*_00_ by the year in which we began using it. The *ab initio* results fitted by *φ*_00_(*r*) come from three sources: (1) at small *r* (1 < *r* < 2.5 bohr), the results of the variational calculation from Komasa [24], (2) at intermediate *r* (3 bohr < *r* < 7 bohr), the SAPT results from Korona et al. [18], (3) at large *r*, the asymptotic constants from the “exact” dispersion coefficients of Bishop and Pipin [22] and the higher order dispersion coefficients determined from the approximate relations presented by Thakkar [29]. The algebraic representation of *φ*_00_(*r*) is a modification of the form given by Tang and Toennies [9]. The representation is the sum of repulsive (*φ*_rep_) and attractive (*φ*_att_) terms:

$$\begin{array}{l} \varphi_{00}\left(r\right)=\{\begin{array}{ll} \varphi_{\text{rep}}\left(r\right)+\varphi_{\text{att}}\left(r\right), & 0.3\leq r/\text{bohr}\;<\infty \\ \varphi_{\text{rep}}\left(0.3\;\text{bohr}\right)+\varphi_{\text{att}}\left(0.3\;\text{bohr}\right), & 0\leq r/\text{bohr}<0.3 \end{array}\\ \varphi_{\text{rep}}\left(r\right)=A\exp\left(a_{1}r+a_{2}r^{2}+a_{-1}r^{-1}+a_{-2}r^{-2}\right),\\ \varphi_{\text{att}}\left(r\right)=-\sum_{n=3}^{8}\frac{f_{2n}\left(r\right)C_{2n}}{r^{2n}}\left[1-\left(\sum_{k=0}^{2n}\frac{{\left(\delta r\right)}^{k}}{k!}\right)\exp\left(-\delta r\right)\right]. \end{array}$$ (1)

Equation (1) includes the factor *f*_2_*_n_*(*r*) that accounts for the relativistic retardation of the dipole-dipole (*n* = 3) term applied over all *r*. This factor changes the behavior of the dipole-dipole term from *r*^−6^ to *r*^−7^ at very large *r*, and it was taken from Jamieson et al. [30]. When the expressions for the retardation of the higher dispersion terms *C*_8_ and *C*_10_ given by Chen and Chung [23] were applied to *φ*_00_, the well depth changed by only 0.0014 K out of 11 K. The resulting changes in the calculated thermophysical properties were much smaller than their uncertainties; thus, we used the approximation *f*_2_*_n_*(*r*) ≡ 1 for *n* > 3. (Note: retardation is included when calculating the thermophysical properties; however, by convention, it is not included when comparing Eq. (1) to the *ab initio* results.) We also considered the adiabatic correction of the helium dimer given by Komasa et al. [28]. The effects of this correction were also much smaller than those from the uncertainties in *φ*(*r*); thus, we omitted this correction.

The definition of *φ*_00_(*r*) in Eq. (1) is broken into two ranges. If this were not done, *φ*_00_(*r*) would have a spurious maximum at very small values of *r*. As indicated in Eq. (1), the break-point was set at 0.3 bohr.

The dispersion coefficients (*C*_6_, *C*_8_, … *C*_16_) in Eq. (1) and Table 1 were held fixed [22, 29]. The values of the remaining parameters in Table 1 (*a*_−2_, *a*_−1_, *a*_1_, *a*_2_, and δ) were determined by fitting *φ*_00_(*r*) to the *ab initio* results. When fitting *φ*_00_ the *ab initio* results were weighted in proportion to the reciprocal of the uncertainty squared, where the uncertainties were taken (when available) from the publications that presented the results. [15, 18, 20, 21, 24].

#### 2.4.2 *φ*_SAPT_

Korona et al. fitted their SAPT results and the QMC values of Ceperley and Partridge [15] to the algebraic expression of Tang and Toennies [9] while holding constant the asymptotic dispersion coefficients of Bishop and Pipin [22]. They included higher order dispersion coefficient determined with combining rules of Thakkar [29] and retardation effects of the *C*_6_ dispersion coefficient as given by Jamieson et al. [30]. Janzen and Aziz [11] calculated the thermophysical properties of helium using *φ*_SAPT_ and they “judged it to be the most accurate characterization of the helium interaction yet proposed.” We believe that *φ*_00_ is more accurate than *φ*_SAPT_ because it uses the recent, accurate variational results of Komasa [24] instead of the earlier short range QMC values of Ceperley and Partridge [15].

#### 2.4.3 *φ*_A_ $\varphi_{A}^{-}$, and $\varphi_{A}^{+}$

In an attempt to ascertain how uncertainties in the interaction energies propagate into the thermophysical properties we constructed alternative potentials which differed in the choice of *ab initio* results, and in the form of the algebraic expression. The first alternative, denoted *φ*_A_, was obtained by fitting the exact same *ab initio* results from [18, 22, 24, 29] as *φ*_SAPT_. The algebraic expression of Tang and Toennies [9] was modified by adding a *a*_3_*r*^3^ to the exponent of the repulsive term, such that *φ*_rep_ = *A* exp(*a*_1_*r* + *a*_2_*r*^2^ + *a*_3_*r*^3^). The additional *a*_3_*r*^3^ term enables *φ*_A_ to fit the *φ*_SAPT_ *ab initio* results within 0.1 % in two regions *r* = 3 bohr and at *r* > 6 bohr where *φ*_SAPT_ [18] deviates from the *ab initio* results slightly greater than 0.1 %.

To obtain $\varphi_{A}^{-}$, we decreased the *ab initio* short-range [15] and long-range [22] results by their claimed uncertainties and decreased the intermediate-range SAPT results by 0.1 % and the long-range dispersion coefficients by 0.08 %. Equation (1) was then re-fitted to obtain $\varphi_{A}^{-}$. We then increased the *ab initio* results by their claimed uncertainties and the intermediate-range SAPT results by 0.1 % and again fitted them to obtain $\varphi_{A}^{+}$.

#### 2.4.4 *φ*_B_

The potential *φ*_B_, uses the CCSD(T) results of van Mourik and Dunning [21] and of van de Bovenkamp and van Duijneveldt [20] instead of the SAPT results of Korona et al. [18] in the intermediate range of 3 bohr < *r* < 7 bohr. To fit these values the algebraic expression of Tang and Toennies [9] was modified again by adding a *a*_−1_*r*^−1^ and *a*_−2_*r*^−2^ to the exponent of the repulsive term, such that *φ*_rep._ = *A* exp(*a*_1_*r* + *a*_2_*r*^2^ + *a*_−1_*r*^−1^ + *a*_−2_*r*^−2^).

### 2.5 Comparison of *φ*_00_, *φ*_SAPT_, *φ*_A_, and *φ*_B_

Table 2 and the lower panel of Fig. 1 display the changes in *φ*(*r*) resulting from alternate choices among the *ab initio* results. The differences between the thermophysical properties calculated using *φ*_00_, *φ*_SAPT_, *φ*_A_, and *φ*_B_ are analogous to the differences between measurements of thermophysical properties conducted in different laboratories using different methods and they are used to estimate the uncertainties of the results for pure ^3^He and pure ^4^He.

Table 3 lists some characteristic properties of the potentials that we have used. They include the well depth *ε*/*k*_B_, the locations of the zero (*σ*) and of the minimum (*r*_m_) of the potential, and the energy of the bound state (*E*_b_) of a pair of ^4^He atoms. Following Janzen and Aziz [31], we estimated the number of Efimov states *N*_E_ from the scattering length and the effective range with the result *N*_E_ = 0.77 ± 0.01 for *φ*_00_. Because *N*_E_ < 1 for all potentials in Table 2, Efimov states are unlikely to exist. A discussion of these properties of the interatomic potential for helium can be found in Ref. [31].

## 3. Numerical Calculations and Their Uncertainties

Here, we outline the steps required to calculate the thermophysical properties of helium from the interatomic potential. We also describe the precautions that were taken to insure that the uncertainties in the results from approximations in statistical mechanics and in the numerical methods were both smaller than the uncertainties results from different choices for *φ*(*r*).

The initial steps of calculating the thermophysical properties that depend upon pairs of helium atoms are all the same. (1) The Schrödinger equation for the scattering of a helium atom at the energy *E* in the potential *φ*(*r*) is separated in spherical coordinates, (2) the radial part of the wave function is expanded in partial waves *ψ_ℓ_*(*r*) of angular momentum *ℓ*, (3) several nodes of the scattered wave are located far from the scattering atom, and (4) the phase shifts *δ_ℓ_* of the scattered wave are determined and (5) summed with appropriate statistics to obtain cross sections. The summations account for large symmetry effects at low temperatures [32]. Thus, separate summations are required for ^3^He and ^4^He and their mixtures when calculating the second virial coefficient and the transport properties. The final step (6) is an integration over energy that is appropriate to the thermophysical property under consideration.

### 3.1 Integration of the Radial Schrödinger Equation

The Schrödinger equation is separated in spherical coordinates and decomposed into angular momentum states to obtain

$$\left(\frac{d^{2}}{dr^{2}}+k^{2}-\frac{\ell \left(\ell +1\right)}{r^{2}}-\frac{2\mu }{\hbar }\varphi \left(r\right)\right)\psi_{\ell }\left(r\right)=0$$ (2)

where *ħ* is Planck’s constant [14] divided by 2π, *µ* is the reduced mass *µ*= (*m*_1_ + *m*_2_)/*m*_1_*m*_2_, *k* = (2*µE*)^1/2^/*ħ* is the wave number, and *E* is the energy of the incoming wave. Equation (2) is integrated to obtain the perturbed wave functions *ψ_ℓ_*(*k*, *r*). The location *r_n_* of the *n*th zero (or node) of the wave function *ψ_ℓ_*(*k*, *r*) was found using a five point Aiken interpolation formula with values of *ψ_ℓ_*(*k*, *r*) near the *n*th node. The integration was performed using Numerov’s method [33] as implemented in [34] and [35]. At each energy, *r_n_* was recalculated using successively smaller step sizes. The calculation was terminated when halving the step size changed *r_n_* less than 10^−9^ × *r_n_*. We verified that the tolerance 10^−9^ × *r_n_* was sufficiently small that further reductions of the step-size did not change the thermophysical properties beyond the tolerances given in Table 6. The final sizes of the integration steps are listed in Table 4.

### 3.2 Calculation of Phase Shifts, *δ_ℓ_*(*k*,*n*)

The relative phase shifts, *δ_ℓ_*(*k*,*n*) of the outgoing partial wave were evaluated from the relation

$$\delta_{\ell }\left(k,n\right)=\arctan\frac{j_{\ell }\left(k,r_{n}\right)}{n_{\ell }\left(k,r_{n}\right)}$$ (3)

where j*_ℓ_*(*k*,*r_n_*) and n*_ℓ_*(*k*,*r_n_*) are the Bessel and Neuman functions for angular momentum quantum number *ℓ* and wave number *k*. In practice, the phase shifts were evaluated at groups of three consecutive nodes. If the phase shift did not change by more than 10^−8^ × *δ_ℓ_*(*k*,*n*) between the first and last of the three nodes, it was assumed that *n* (and *r_n_*) were sufficiently large that additional effects of the potential were negligible, and the calculation was terminated. Otherwise, the calculation was continued to larger values of *r*, and the test was repeated. We verified that the tolerance 10^−8^ × *δ_ℓ_*(*k*,*n*) is consistent with the uncertainties of the thermophysical properties listed in Table 6.

### 3.3 Calculation of the Second Virial Coefficient, *B*(*T*)

The second virial coefficient was obtained by adding two or three terms; the first term is a thermal average *B*_th_(*T*), the second term is that of an ideal gas *B*_ideal_(*T*), and the third term is the bound state term *B*_bound_(*T*), which applies to ^4^He, but not to ^3^He because a bound state exists only for ^4^He.

#### 3.3.1 The Thermal Average Term *B*_th_(*T*)

The thermal average term *B*_th_(*T*) is

$$B_{\text{th}}=\int_{0}^{\infty }k\exp\left(-\frac{k^{2}}{k_{B}T}\right)\sum_{\ell =0}^{\infty }\left(2\ell +1\right)\delta_{\ell }\left(k,n≃\infty \right)dk$$ (4)

where *k*_B_ is the Boltzmann constant, and *δ_ℓ_*(*k*,*n* ≃ ∞) is the phase shift at large enough separation that the potential no longer perturbs the outgoing wave function [32, 36].

Equation (4) contains both a sum and an integral with the limits 0 and ∞. Truncating the sum and the integral at a finite upper bound is a potential source of error. At each value of *k*, the sum was computed until the addition of six phase shifts did not change the sum by more than 10^−8^ of its value. At the lowest energies, this condition was met after adding seven phase shifts; at the highest energies, hundreds of phase shifts were added.

At this step of the calculation, symmetry effects are incorporated. The unweighted sum [Eq. (4)] is carried out over all values of *ℓ* only when calculating the interaction virial coefficient for mixtures of ^3^He and ^4^He, because these atoms are distinguishable and follow Boltzmann statistics. For pure ^3^He and ^4^He, weighted sums are performed over the even and odd values of *ℓ* using the formulas

$$\begin{array}{c} \sum_{\text{BE}}=\left[\frac{s+1}{2s+1}\right]\sum_{\text{even}}+\left[\frac{s}{2s+1}\right]\sum_{\text{odd}}\;\\ \sum_{\text{FD}}=\left[\frac{s+1}{2s+1}\right]\sum_{\text{odd}}+\left[\frac{s}{2s+1}\right]\sum_{\text{even}}\; \end{array}$$ (5)

where *s* is the spin quantum number (0 for ^4^He; ½ for ^3^He), BE stands for Bose-Einstein statistics for bosons (^4^He), and FD stands for Fermi-Dirac statistics for fermions (^3^He). Details on this calculation can be found in Ref. [32].

The integral in Eq. (4) was evaluated using a standard integration routine, DQAGI [37]. This routine is designed for semi-infinite or infinite intervals and automatically uses nonlinear transformation and extrapolation to achieve user-specified absolute and relative tolerances for a user-specified function. The relative error was set to 10^−8^. If the integrator could not achieve this accuracy, an error message would have been reported the problem.

#### 3.3.2 The Ideal-Gas and Bound State Terms *B*_ideal_(*T*) and *B*_bound_(*T*)

The ideal-gas contribution *B*_ideal_(*T*) is negative for BE and positive for FD, and zero for Boltzmann statistics as given by

$$B_{\text{ideal}}=\pm N_{A}2^{-5/2}\;\lambda^{3}$$ (6)

where *N*_A_ is the Avagodro constant and *λ* ≡ [*ħ*/(*µk*_B_*T*)]^1/2^ is the “thermal wavelength.” The ideal-gas term is important only at low temperatures; it is 1/10 of *B*(*T*) at 5 K and 1/100 of *B*(*T*) at 75 K. The ideal-gas term is a function of fundamental physical constants and the resulting standard uncertainty is on the order of 10^−6^.

For ^4^He, the bound state term *B*_bound_(*T*) is

$$B_{\text{bound}}=-N_{A}2^{-3/2}\;\lambda^{3}\left(e^{E_{b}/k_{B}T}-1\right)$$ (7)

where *E*_b_ is the energy of the bound state. The bound state term is 1/1000 of *B*(*T*) at 3 K and 1/100 of *B*(*T*) at 0.4 K. *E*_b_ was determined from integrating the Schrödinger equation; thus, it depended upon the integration step size. Decreasing step sizes were used until consecutive values of *E*_b_ differed by less than 10^−6^ × *E*_b_. This numerical uncertainty is much smaller than the 18 % difference between *E*_b_ determined from $\varphi_{A}^{-}$ and that determined from $\varphi_{A}^{+}$ (Table 3).

The sum of the numerical uncertainties in the calculation of *B*(*T*) is at most 10^−5^ × *B*(*T*). This is insignificant compared with the uncertainty of *B*(*T*) which arises from the uncertainty of the potential *φ*(*r*). For example, the uncertainty of *B*(*T*) resulting from the uncertainty of *φ*(*r*) is 0.0022 × *B*(*T*) at 300 K; the relative uncertainties at other temperatures are listed in Table 6.

### 3.4 Calculation of the Transport Properties

In order to calculate the transport properties, we used the numerical methods outlined above to obtain the phase shifts as functions of the wave number and angular momentum quantum number. Then we computed the sums over the phase shift that determine the quantum cross sections, *Q*^(1)^, *Q*^(2)^, *Q*^(3)^….*Q*^(n)^, etc. [38]. The cross sections were integrated with respect to energy to obtain the temperature-dependent collision integrals. Finally, the transport properties were calculated using the appropriate combinations of the collision integrals.

#### 3.4.1 Calculation of the Quantum Cross Sections *Q*^(n)^

The quantum cross sections are functions involving the sums of the phase shifts that depend upon the symmetry of the interacting atoms. The sums over the even and the odd values of *ℓ* are needed separately:

$$\begin{array}{c} Q_{\text{odd}}^{\left(1\right)}=\frac{4\pi }{k}\sum_{\ell =1,3,5\ldots }^{\infty }\left(2\ell +1\right)\sin^{2}\delta_{\ell }\\ Q_{\text{even}}^{\left(1\right)}=\frac{4\pi }{k}\sum_{\ell =0,2,4\ldots }^{\infty }\left(2\ell +1\right)\sin^{2}\delta_{\ell } \end{array}$$ (8)

and then weighted sums are computed. To evaluate *Q*^(1)^ for Bose-Einstein (BE) or Fermi-Dirac (FD) statistics the sums are weighted with the spin-dependent quotients, as shown in Eq. (5). As for the case of the second virial coefficient, the sums in Eq. (8) extend to *ℓ* = ∞. The sum was continued until the addition of six more phase shifts changed the cross section by less than 10^−8^ of its value. Cross sections with moments up to *n* = 6 are required to calculate the collision or omega integrals used in the higher order approximations for the transport properties. The equations for these calculations are given by Ref. [38].

#### 3.4.2 Calculation of the Collision Integrals *Ω*^(^*^n,s^*^)^

The reduced collision integrals were evaluated from the equation

$$\begin{array}{r} \Omega^{\left(n,s\right)★}\left(T^{★}\right)={\left\{\left(s+1\right)!T^{★\left(s+2\right)}\right\}}^{-1}\\ \times \int_{0}^{\infty }Q^{\left(n\right)★}\left(E^{★}\right)e^{-E^{★}/T^{★}}E^{★\left(s+1\right)}\;dE^{★} \end{array}$$ (9)

where the superscript ^★^ indicates that both the energy and the temperature were scaled by the well-depth of *φ*_00_ and *Q*^(^*^n^*^)★^ was scaled by the value *Q*^(^*^n^*^)★^ for a rigid sphere of radius *r*_m_, the location of the minimum of *φ*_00_ (Table 3; See Ref. [32]).

In order to evaluate of Eq. (9), the quantum cross sections *Q*^(^*^n^*^)★^ must be calculated at each energy *E* used for the quadrature. We calculated a table of *Q*^(^*^n^*^)★^ as a function of *E*^★^ and used a 5 point Aiken interpolation to determine values of *Q*^(^*^n^*^)★^ between tabulated values. The intervals in the table were determined such that the interpolated values had a uncertainty of less that 10^−6^ × *Q*^(^*^n^*^)★^. Equation (9) was integrated using the automated quadrature routine DQAGI [37], discussed in Sec. 3.1.3, with the tolerance set to 10^−8^. The numerical methods used to calculate the collision integrals yielded results with a relative uncertainty of less than 10^−5^.

#### 3.4.3 Calculation of the Transport Properties From the Collision Integrals

**Fig. 2:**
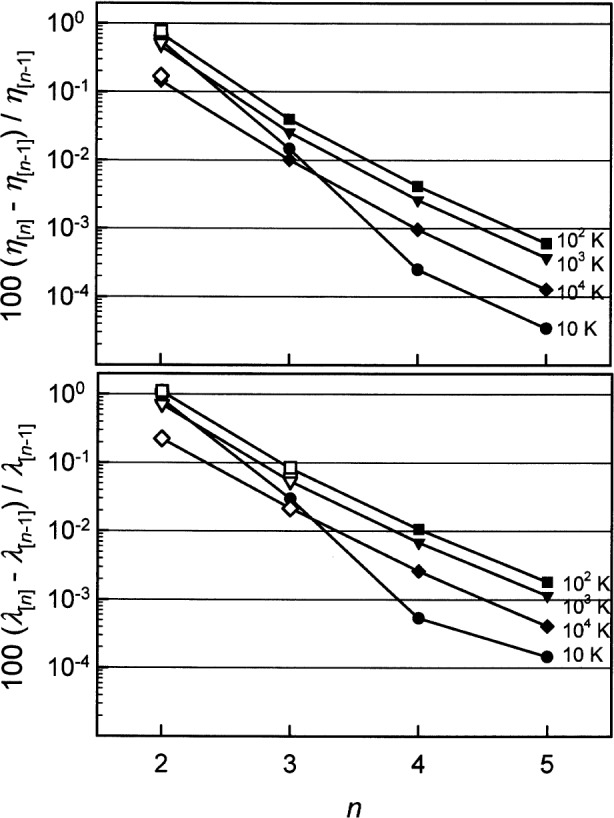
Asymptotic approach of viscosity and thermal conductivity of ^4^He as a function of increasing order of approximation along the four indicated isotherms. The open symbols are for the equimolar mixture of ^3^He and ^4^He.

The transport properties of dilute gases are calculated using combinations of the collision integrals in approximations of increasing complexity and accuracy. The viscosity and thermal conductivity of pure ^3^He and ^4^He were calculated to the 5th order approximation [39]. The equimolar mixture thermal conductivity [40] and thermal diffusion factors [41] were calculate to the 3rd order, and the diffusion coefficient and mixture viscosity were calculated to 2nd order. Figure 2 shows the effects of truncating the order of the calculation. The changes in *η* and *λ* for ^4^He and equimolar mixtures of ^4^He and ^3^He are compared at four temperatures upon increasing order of the approximation. The calculations converge very well; 2nd to 3rd order results in less than a 0.1 % change, 3rd to 4th order results in less than a 0.01 % change, and 4th to the 5th order less than 0.001 %. The behavior of the other transport properties (*η* and *λ* for pure ^3^He, *D*_12_, and *α*_T_) is similar to that shown in Fig. 2. Figure 2 shows that the change in *η* and *λ* of the equimolar mixture from 1st to 2nd order, is very close to that of pure ^4^He. These results show that only calculations of the 2nd order contribute any significant uncertainty to the calculated properties. From these observations, we conclude that the relative uncertainty of *η* and *D*_12_ for the equimolar mixture ranges from 0.01 % to 0.04 % in the temperature range 10 K ≤ *T* ≤ 10^4^ K. Figure 2, together with the equivalent figure for pure ^3^He, suggest that, at *T* > 100 K, the accuracy of the calculated *η* and *D*_12_ for the equimolar mixture might be improved if one extrapolated from 2nd order to 5th order by following the curves for pure ^4^He and ^3^He.

The rapid reduction of the uncertainty of the calculated viscosity with increasing order of approximation is not sensitive to *φ*(*r*); Viehland et al. [39] obtained similar results for the viscosity of rigid atoms that interact via (12-6) Lennard-Jones potentials.

### 3.5 Interpolation as a Function of Temperature

The tables in Appendix A list values of the second virial coefficient, the transport properties, and their first derivatives as functions of temperature. The temperature intervals were chosen so that the errors from linear interpolation would be smaller than the uncertainties propagated from the uncertainties of the interatomic potential. Table 5 lists bounds of the interpolation errors, and the unweighted average over the entire temperature range. Below 10 K, the interpolation errors increase because the temperature derivatives of the properties increase.

### 3.6 Classical Calculation

**Fig. 3:**
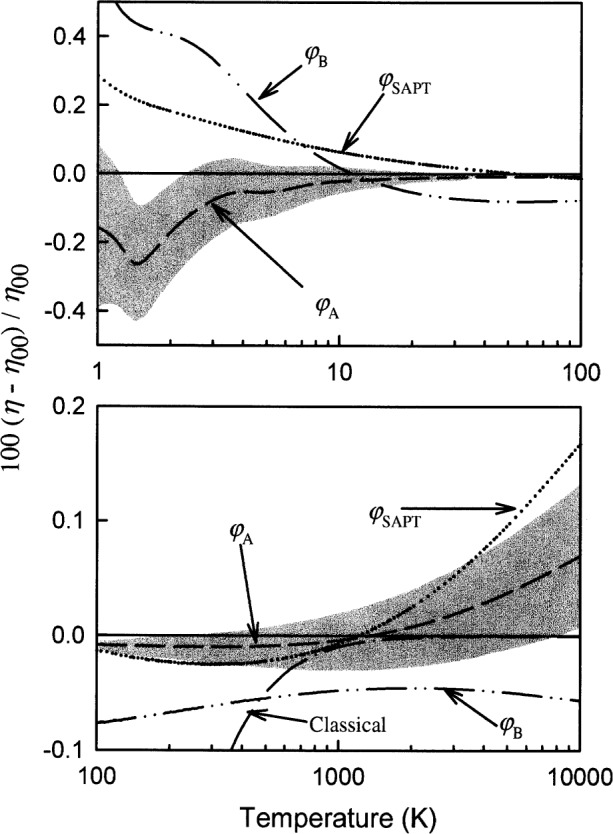
Fractional deviation of the calculated viscosity using the considered potentials. The base line is the viscosity calculated to the 5th approximation for ^4^He from *φ*_00_. The other curves are identified in the figure. The shaded region around the curve for *φ*_A_ shows how $\varphi_{A}^{-}$ and $\varphi_{A}^{+}$ vary the predicted viscosity. Also shown is the classically calculated viscosity asymptotically approaching the quantum values with increasing temperature.

**Fig. 4:**
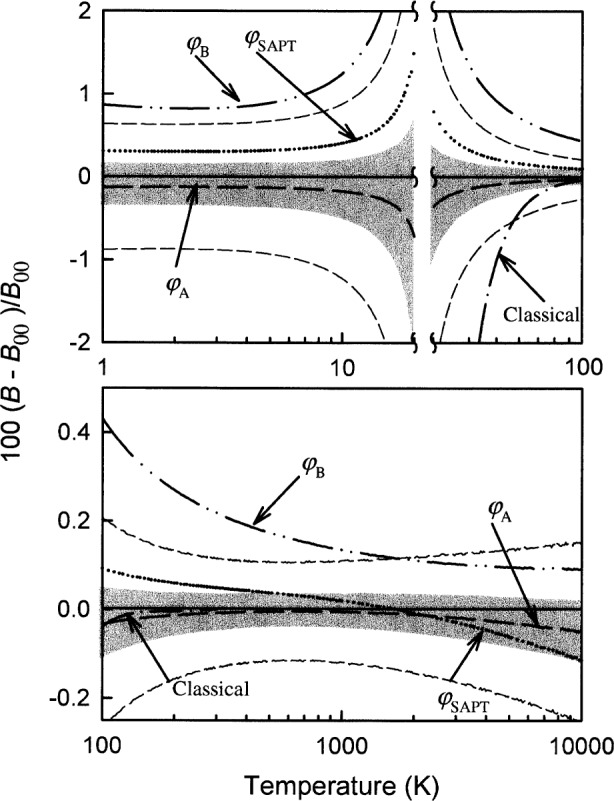
Fractional deviation of the calculated second virial coefficient using the considered potentials. The base line is *B*_00_ calculated for ^4^He using *φ*_00_. The other curves are as identified in the figure. The shaded region around the curve for *B*_A_ shows how $\varphi_{A}^{-}$ and $\varphi_{A}^{+}$ influence the predicted *B*. Also shown is the classically calculated value for *B*(*T*) asymptotically approaching the quantum values with increasing temperature.

We made an important check of the entire calculation of each thermophysical property. To do so, we performed the relatively simple classical calculation [32] which is valid at high temperatures where the ratio of the de Broglie wavelength *h*(2π*mkT*)^−1/2^ to atomic diameter *σ* is much less than 1. Figures 3 and 4 show that the classical calculations of the viscosity and of the second virial coefficient asymptotically approach the quantum results.

## 4. Uncertainties of the Thermophysical Properties From the Uncertainty of the Potential

We now evaluate how the uncertainty of the *ab initio* values of *φ*(*r*) propagates into the uncertainty of calculated thermophysical properties. To do so, we calculated the properties with each of the potentials discussed in Sec. 2 and we plotted the results as deviations from the results obtained for *φ*_00_(*r*). Figure 3 shows these deviations for the viscosity of ^4^He. In Fig. 3, the width of the shaded band surrounding the curve *φ*_A_ spans the range of results obtained with $\varphi_{A}^{-}$ to those obtained with $\varphi_{A}^{+}$. Similar bands could have been placed about the results from those obtained with *φ*_00_, *φ*_B_, and *φ*_SAPT_; they were omitted for clarity.

We took the differences in the alternative potentials as an accurate estimate of the uncertainty in *φ*_00_. By comparing the properties calculated from each alternative potential, we estimated the actual uncertainty propagated into each reported thermophysical property. Figure 3 shows that as the temperature is increased from 1 K to 10 K, the relative uncertainty of the viscosity *u*_r_(*η*) of ^4^He decreases from 0.4 % to 0.1 %. In this temperature range, the discrepancies among the potentials are comparable to the uncertainty of each potential, as indicated by the width of the shaded band. In the range 10 K < *T* < 1000 K, the difference between the results obtained using *φ*_00_ and the results obtained with *φ*_B_ and *φ*_SAPT_ lead us to conclude that *u*_r_(*η*) is approximately 0.08 %. If the discrepancies between the *ab initio* results around 4.0 bohr could be resolved, then *u*_r_(*η*) would be reduced by nearly a factor of three in this temperature range. In the range 1000 K < *T* < 10^4^ K, we also conclude *u*_r_(*η*) ≈ 0.08 %. In this temperature range, the results from *φ*_00_ and *φ*_B_ are more reliable than the results from *φ*_A_ and *φ*_SAPT_ having been fit to the short-range variational calculations of Komasa [5] as discussed in Sec. 2, above.

The relative uncertainty of the second virial coefficient *u*_r_(*B*) of ^4^He can be judged from Fig. 4. In the range 1 K < *T* < 10 K, *u*_r_(*B*) ≈ 1 %. At *T* ≈ 23.4 K, *B*(*T*) passes through zero. There, *u*_r_(*B*) diverges; however, the uncertainty of *B*, *u*(*B*) ≈ 0.3 cm^3^·mol^−1^. In the range 100 K < *T* < 10^4^ K, *u*_r_(*B*) of ^4^He gradually declines from 0.4 % to 0.1 %.

The uncertainties for each property are summarized in Table 6 from comparisons similar to those provided in Figs. 3 and 4 and described in the preceding paragraphs. These uncertainties are much lower than those from measurements; thus, the corresponding values of the properties listed in the Appendices can be used as standards.

## 5. Results

The results of the present calculations for ^4^He, ^3^He, and their equimolar mixture are listed in Tables A1, A2, and A3 in Appendix A. These tables contain the second virial coefficient *B* for the pure species and the interaction second virial *B*_12_ where $B_{\text{mix}}=x_{1}^{2}B_{11}+2x_{1}x_{2}B_{12}+x_{2}^{2}B_{22}$. The zero-density viscosity, thermal conductivity and their equimolar mixture. The diffusion coefficient at 101.325 kPa (one atmosphere), and the thermal diffusion factor. Derivatives with respect to temperature are provided to facilitate interpolation and for use in calculating acoustic virial coefficients. The tables for pure ^4^He and ^3^He contain the self-diffusion coefficient calculated without symmetry effects (Boltzmann statistics), and the thermal diffusion factor of a mixture of 99.999 % ^4^He or ^3^He respectively. The tables span the temperature interval 1 K ≤ *T* ≤ 10^4^ K. The highest temperature is well below the first excited state of helium (2 × 10^5^ K) and well below 2.91 × 10^5^ K, the highest value of the *ab initio* results used to determine *φ*_A_. In order to calculate the thermophysical properties between the tabulated temperatures, we recommend interpolation using the cubic polynomial *f*(*T*) such that

$$\begin{array}{l} f\left(T\right)=a\left(T-T_{1}\right)+b\left(T-T_{2}\right)+\left\{c\left(T-T_{1}\right)+d\left(T-T_{2}\right)\right\}\left(T-T_{1}\right)\left(T-T_{2}\right)\\ \begin{array}{cc} a=f\left(T_{2}\right)/\Delta T & c=\left\{f^{\prime }\left(T_{2}\right)/\left(\Delta T\right)\right\}-\left\{\left(a+b\right)/{\left(\Delta T\right)}^{2}\right\}\\ b=f\left(T_{1}\right)/\Delta T & d=\left\{f^{\prime }\left(T_{2}\right)/{\left(\Delta T\right)}^{2}\right\}-\left\{\left(a+b\right)/{\left(\Delta T\right)}^{2}\right\}, \end{array} \end{array}$$ (10)

where *f*′ = d*f*/d*T* and Δ*T* = *T*_2_ − *T*_1_. The calculated values listed in the Tables are accurate to the uncertainties discussed in Sec. 4. Equation (10) contributes an additional uncertainty from the interpolation discussed as in Sec. 3.

## 6. Comparison With Measurements

In this section we compare the values of the thermophysical properties calculated using *φ*_00_ with the best experimental values. In nearly every case, the experimental values agree with the calculated values within their combined uncertainties, and the calculated properties have the smaller uncertainties.

### 6.1 Second Virial Coefficient

**Fig. 5:**
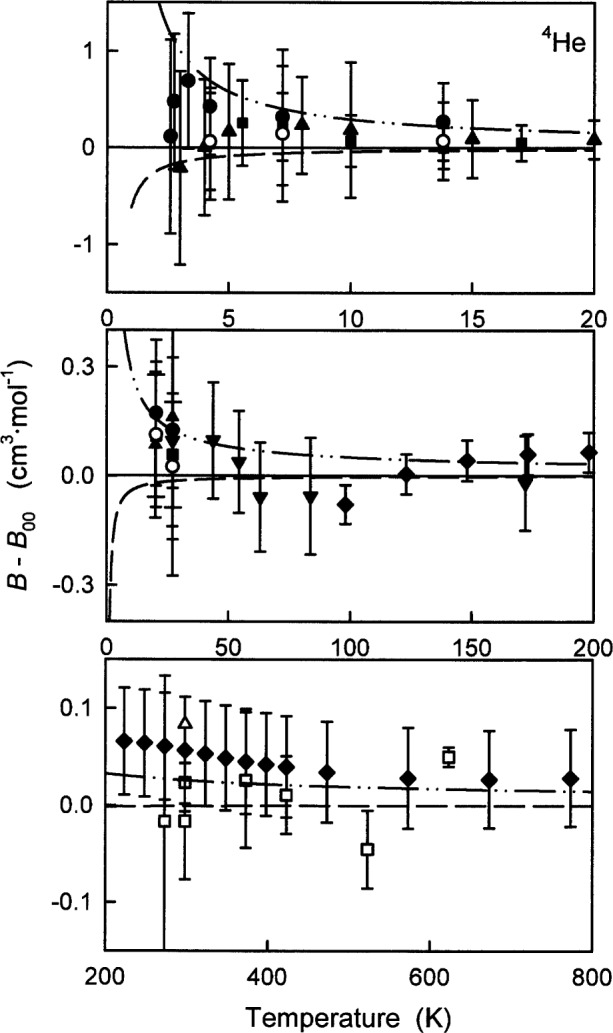
Deviations of *B*_exp_ of ^4^He from *B*_00_ calculated using *φ*_00_. Key: ● Ref. [42]; ○ Ref. [42] adjusted by Ref. [43]; ■ Ref. [43]; ▲ Ref. [43]; ▼ Ref. [44]; ♦ Ref [45]; □ Ref. [46]; △ Ref. [8] Eqs. (37) and (38); — — Values of *B*_A_(*T*) calculated using *φ*_A_; — • • — *B*_B_(*T*) calculated using *φ*_B_.

Figure 5 displays the deviations of various experimental values of *B*(*T*) for ^4^He from *B*_00_(*T*) calculated using *φ*_00_. The dashed curves in Fig. 5 represent the values of *B*_A_(*T*), calculated using *φ*_A_, and the dash-dot-dot curves the values of *B*_B_(*T*), calculated from *φ*_B_. Also shown in Fig. 5 and summarized in Table 7, are measured values of *B*(*T*) along with their reported uncertainties. In nearly every case, *B*_00_(*T*) agrees with the experimental values within the uncertainties of the experimental values. The maximum uncertainties of *B*_00_(*T*) are estimated by comparing the variances with *B*_A_(*T*) and *B*_B_(*T*). These uncertainties are much smaller than the experimental uncertainties (see the dash-dot-dot curve in Fig. 5.).

At very low temperatures *B*(*T*) is sensitive to the shape of the potential well. Figure 5 shows that the lower well depth of *φ*_00_ predicted by Korona et al. [18] reproduces the low temperature measurements better than the shallower well depth predicted by Van de Bovenkamp and van Duijneveldt [20] and by Van Mourik and Dunning [21]. To further strengthen this argument, it is known that the low temperature second virial measurements have not been corrected for contributions from the third virial coefficient *C*(*T*). For ^4^He [42], the size of this “third virial correction” can be seen in the top panel of Figure 5. In that panel, the solid circles show the values *B*(*T*) before they were corrected in Ref. [43], and the open circles show the values after the correction. The correction for *C*(*T*) lowers the second virial values bringing them further in line with *B*_00_(*T*) and away from *B*_B_(*T*) indicating a preference for the lower well depth.

Table 7 provides two numerical measures of the differences between experimental values of *B*(*T*) and those calculated using *φ*_00_. One measure is the mean of the absolute values of the differences *B*_exp_ − *B*_00_ and the second is the range of these differences. The final column of Table 7 lists the range of the uncertainties reported by the experimenters. In nearly all cases the experimental uncertainties exceed the differences *B*_exp_ − *B*_00_.

**Fig. 6:**
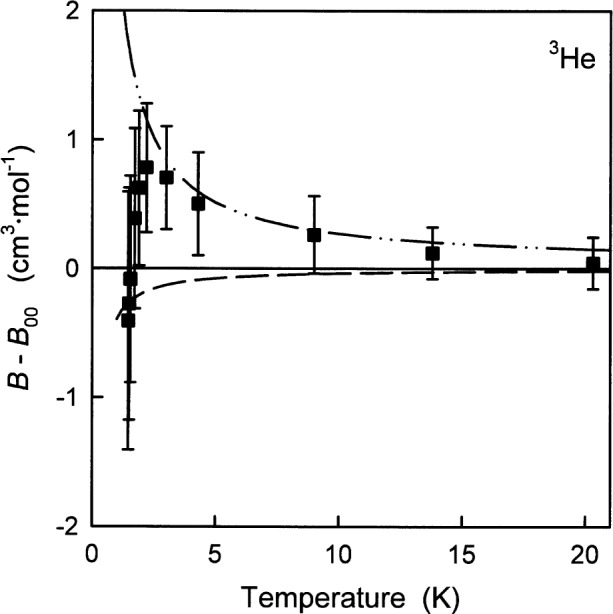
Deviation of *B*_exp_ of ^3^He from *B*_00_ calculated using *φ*_00_(*r*). ■ Ref. [47]; — — Values of *B*_A_ calculated using *φ*_A_; — • • — *B*_B_ calculated using *φ*_B_.

Figure 6 compares *B*_exp_(*T*) of ^3^He, deduced from the measurements of Matacotta et al. [47], with *B*_00_(*T*). There is an obvious trend in the deviations which is larger than the experimental uncertainties below 5 K. Probably, the trend would be removed if *B*_exp_(*T*) was corrected for the for effects of the third virial coefficient of ^3^He [48] as discussed above for ^4^He [42].

### 6.2 Viscosity

**Fig. 7:**
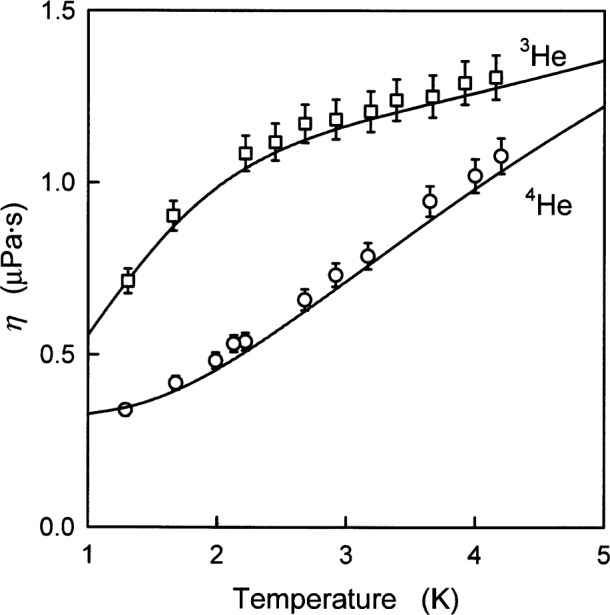
The viscosity of ^3^He and ^4^He at low temperature. Experimental values: □ for ^3^He and ○ for ^4^He from Ref. [49], ±5 % uncertainty bars are shown. The curves are *η*_00_ calculated using *φ*_00_.

**Fig. 8:**
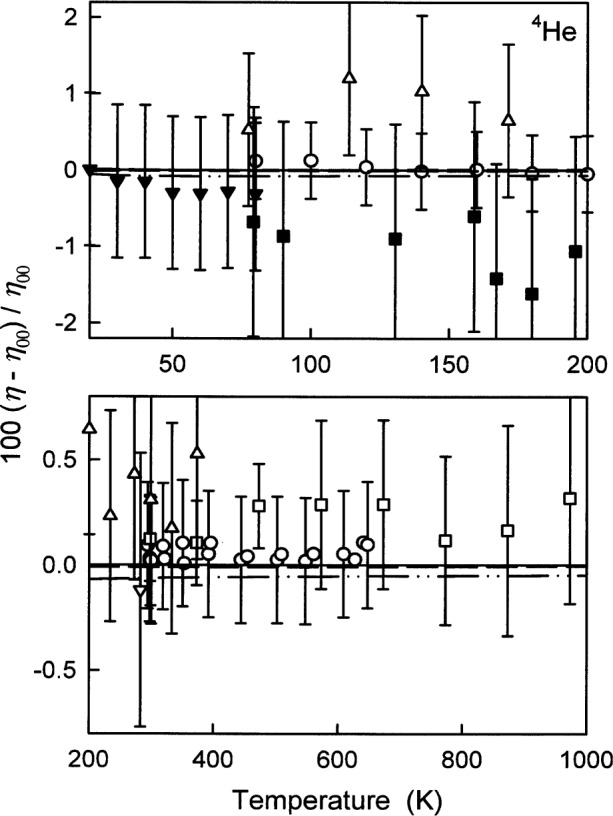
Relative deviations of *η*_exp_ of ^4^He from *η*_00_ calculated using *φ*_00_(*r*). Experimental values: ● Ref. [51]; ■ Ref. [58]; Δ Ref. [54]; ▲ Ref [56] (smoothed); ○ Ref. [52]; □ Ref. [50]; ∇ Ref. [61]; — — Values of *η*_A_ calculated using *φ*_A_; — • • — *η*_B_ calculated using *φ*_B_.

Figures 7 and 8 and Table 8 compare the zero-density viscosity *η*_00_, calculated using *φ*_00_, with measured values from many sources. The experimental results are typically reported at 101.325 kPa where the density dependence is negligible in comparison with experimental uncertainties. Figure 7 shows the viscosity of ^3^He and ^4^He at low temperatures where the large quantum effects lead to important differences between the isotopes. The *η*_00_(*T*) values are in good agreement with the measurements of Becker et al. [49].

Figure 8 displays the fractional deviations of various values of *η*_exp_ of ^4^He from *η*_00_. In nearly every case they are smaller than the uncertainties provided by the experimenters. In Fig. 8, the barely visible dashed curve represents *η*_A_ calculated using *φ*_A_, and the dash-dot-dot curve *η*_B_, calculated from *φ*_B_. The differences between these curves are a measure of the *ab initio* uncertainties which are much smaller than the reported experimental uncertainties.

### 6.3 Thermal Conductivity

**Fig. 9:**
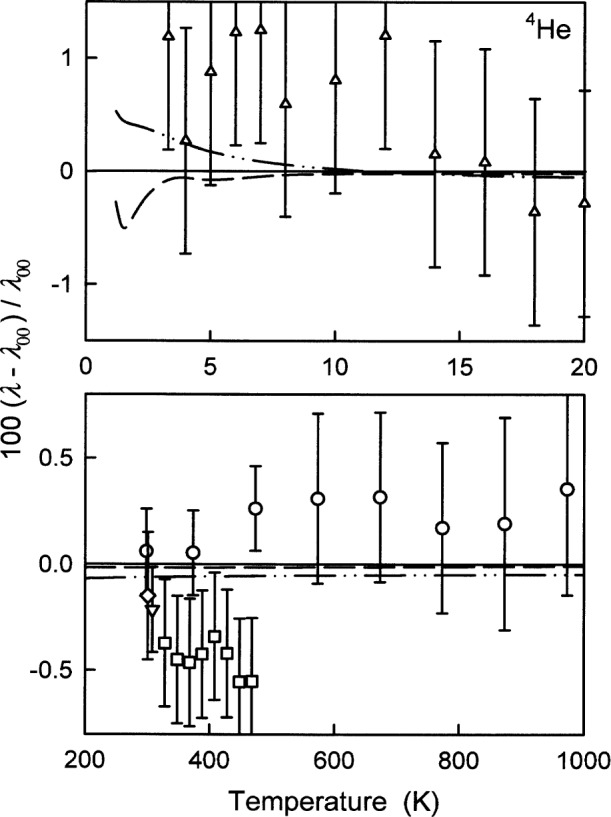
Deviations of *λ*_exp_ from *λ*_00_ calculated using *φ*_00_ for ^4^He. Key: ○ Ref. [50]; □ Ref. [62]; Δ Ref. [65]; ∇ Ref. [64]; ◇ Ref [66]; — — *λ*_A_ calculated using *φ*_A_; — • • — *λ*_B_ calculated using *φ*_B_.

Figure 9 and Table 9 compare the values of the zero-density thermal conductivity of ^4^He calculated using *φ*_00_ with measured values from several sources. The experimental thermal conductivities are typically reported at 101.325 kPa, however the density dependence is negligible compared to the experimental uncertainties. As it was the case for *B* and *η*, most of the values of *λ*_exp_ differ from *λ*_00_ by an amount comparable to the uncertainty of the measurements. The differences between the values *λ*_00_, *λ*_A_, and *λ*_B_ calculated using *φ*_00_, *φ*_A_, and *φ*_B_ are much smaller than the uncertainties of the measurements. Table 9 lists the root mean square of the relative differences Δ*λ*/*λ*_00_ ≡ (*λ*_exp_ − *λ*_00_)/*λ*_00_ and the range of these relative differences. The final column of Table 9 lists the range of the uncertainties reported by the experimenters.

### 6.4 Diffusion Coefficient *D*_12_(*T*)

**Fig. 10:**
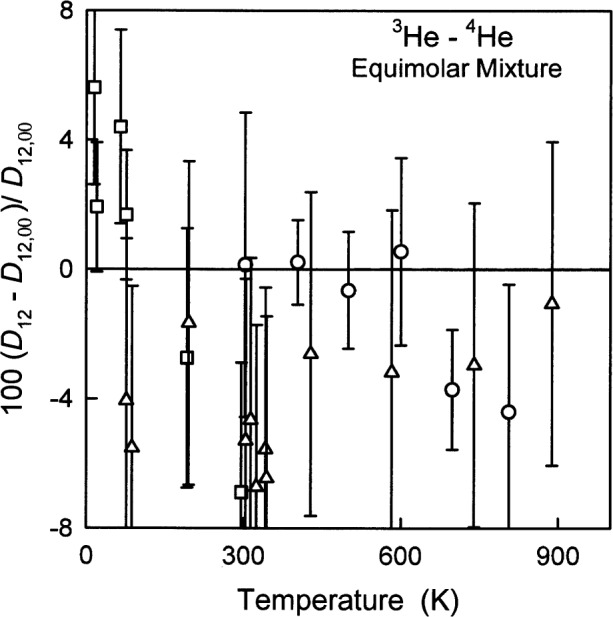
Fractional deviations of *D*_12,exp_ from *D*_12,00_ calculated using *φ*_00_, for an equimolar mixture of ^3^He and ^4^He. Key: ○ Ref. [67]; □ Ref. [68]; Δ Ref. [69]; — — Values of *D*_12,A_ calculated using *φ*_A_; — • • — *D*_12,B_ calculated using *φ*_B_.

Figure 10 and Table 10 compare the values of the mutual diffusion coefficient for an equimolar mixture of ^3^He and ^4^He at one atmosphere (101325 Pa). Figure 10 shows the deviations of *D*_12,exp_ taken from three sources, from *D*_12,00_, where *D*_12,00_ was calculated using *φ*_00_. Because the diffusion coefficient is difficult to measure, the uncertainties of the experimental values are comparatively large; therefore, the relative deviations of the values calculated using *φ*_A_ and *φ*_B_ are not visible in Fig. 10. The difference in *D*_12_ on going from the first to the second order approximation is practically the same as seen for the viscosity in Fig. 2. Table 10 lists the root-mean-square of the relative differences Δ*D*/*D*_00_ ≡ (*D*_exp_ − *D*_00_)/*D*_00_ and the range of these relative differences as well as the range of the uncertainties reported by the experimenters.

### 6.4 Thermal Diffusion Factor *α*_T_

**Fig. 11:**
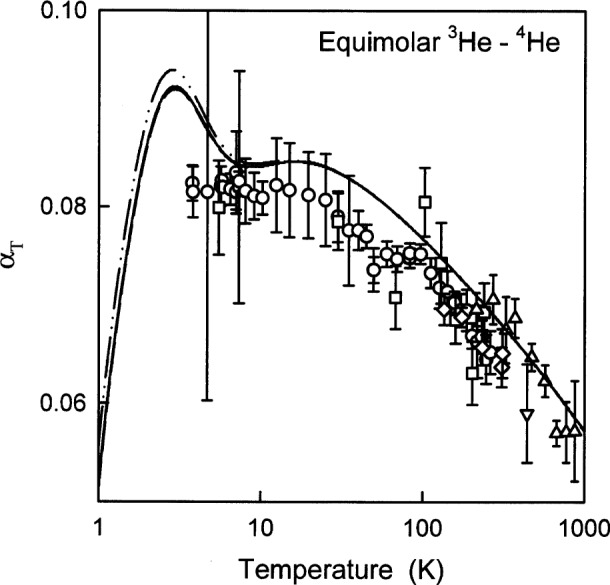
Thermal diffusion factor *α*_T_ as a function of temperature for an equimolar mixture of ^3^He and ^4^He. The solid curve represents *α*_T,00_ calculated using *φ*_00_. Key: ○ Ref. [71]; □ Ref. [72]; Δ Ref. [73]; Δ Ref. [74]; ◇ Ref [75]; — — Values of *α*_T,A_ calculated using *φ*_A_; — • • — *α*_T,B_ calculated using *φ*_B_.

The thermal diffusion factor *α*_T_ is a complicated function of temperature and concentration and only a few, relatively inaccurate measurements are available. Figure 11 compares *α*_T,exp_ for an equimolar mixture of ^3^He and ^4^He to the *α*_T,00_ values calculated from *φ*_00_. The values of *α*_T,A_ and *α*_T,B_ calculated from *φ*_A_ and *φ*_B_ are also shown, only differing at low temperatures. In the first-order approximate calculation of the transport properties, *α*_T_ is identically zero; thus, we compared the second-order transport-theory results to the third-order results to estimate the uncertainties of the *ab initio* results from truncating the transport theory. Going from the second to third order increased *α*_T_ by 0.56 % at 10 K and by 0.36 % at 10,000 K. The thermal diffusion factor is very difficult to measure the typical relative uncertainties are 4 % to 8 %. Owing to the experimental difficulties, the calculated values would be more accurate than any experimentally determined value.

## 7. Conclusion

We have reviewed the recent *ab initio* calculations of *φ*(*r*) for helium. We represented one of the most accurate *ab initio* values of *φ*(*r*) by the algebraic expression *φ*_00_(*r*) and we estimated its uncertainty by comparing the various *ab initio* calculations. For the thermophysical properties, the most significant uncertainties occur near 4.0 bohr. Using *φ*_00_(*r*), we calculated *B*, *η*, *λ*, *D*_12_, and *α*_T_. The numerical methods used in these calculations contributed negligible uncertainty to the results. In all cases, the uncertainties of the calculated thermophysical properties propagated from the uncertainties in *φ*_00_(*r*) were much less than the uncertainties of published measurements. Therefore, the calculated values should be used as standard reference values.

The large number of recent *ab initio* calculations of *φ*(*r*) demonstrate that this is an active field of research. In the near future, *ab initio* calculations will surely reduce the uncertainty of *φ*(*r*) near 4.0 bohr, further reducing the uncertainties in the calculated properties. Improved *ab initio* calculations of the molar polarizability of helium and of the dielectric virial coefficients are also under way. These may well lead to an *ab initio* standard of pressure based on measurements of the dielectric constant of helium near 273.16 K [70].

## Figures and Tables

**Table 1:** Selected *ab initio* values of *φ(r).* (1 bohr = 0.052 917 721 nm)

	*φ*(3.0 bohr)/K	*φ*(4.0 bohr)/K	*φ*(5.6 bohr)/K	Remarks
Ceperley and Partridge [15]	3800 ± 100			“exact” QMC
Anderson et al. [16]	3812 ± 96.0		−11.01 ± 0.10	“exact” QMC
Klopper and Noga [17]		294.5 292.6	−10.68 −11.00	corrected to FCI
Korona et al. [18]	3759.959 ± 11.3	291.64 ± 0.9	−11.06 ± 0.03	SAPT
Komasa [24]	3768.469	292.784	−10.947 −10.978	(1200 term) (2048 term) upper bound
Gdanitz [19]	3768.813 3768.0 ± 0.8	293.025 292.7 ± 0.4	−10.947 −11.05 ± 0.10	extrapolated to ∞ basis set
van de Bovenkamp and Duijneveldt [20]		293.48 292.72 ± 0.02	−10.95 −10.99 ± 0.02	corrected to FCI
van Mourik and Dunning [21]		293.498 292.578	−11.00 ± 0.03 −10.99	corrected to FCI

**Table 2:** Parameters for Eq. (1) in atomic units (1 bohr = 1 Bo = 0.052 917 721 nm, 1 hartree = 1 Ha = 3.157 746 5 × 10^5^ K)

Property (unit)	*φ*_00_	*φ*_A_	$\varphi_{A}^{+}$	$\varphi_{A}^{-}$	*φ*_B_	*φ*_SAPT_
10^−6^ A (K)	2.83379199	2.02311	2.03130	2.01529	3.12631	2.07436426
*a*_1_ (Bo^−1^)	−1.986231822	−1.84827	−1.85059	−1.84616	−2.01639	−1.88648251
10^2^ *a*_2_ (Bo^−2^)	−5.034284240	−7.55879	−7.50314	−7.60470	−4.67475	6.20013490
10^3^ *a*_3_ (Bo^−3^)	0.0	1.82924	1.71078	1.93491	0.0	0.0
*a*_−1_ (Bo)	−0.3514929118	0.0	0.0	0.0	−0.47972	0.0
*a*_−2_ (Bo^2^)	0.1101468439	0.0	0.0	0.0	0.16755	0.0
*δ* (Bo^−1^)	2.00788607	2.03451	2.02137	2.04780	2.01997	1.94861295
*C*_6_ (Ha·Bo^−6^)	1.46097780	1.46098	1.45981	1.46215	1.46098	1.46097780
10^−1^ *C*_8_ (Ha·Bo^−8^)	1.4117855	1.41179	1.41066	1.41291	1.41179	1.4117855
10^−2^ *C*_10_ (Ha·Bo^−10^)	1.83691250	1.83691	1.83544	1.83838	1.83691	1.83691250
a10^−3^ *C*12 (Ha·Bo^−12^)	3.265	3.265	3.262	3.268	3.265	3.265
a10^−4^ *C*_14_ (Ha·Bo^−14^)	7.644	7.644	7.638	7.650	7.644	7.644
a10^−6^ *C*_16_ (Ha·Bo^−16^)	2.275	2.275	2.273	2.277	2.275	2.275

a Calculated using combining rules of Thakkar [29]

**Table 3:** Properties of the fitted helium potentials. (1 Å = 10^−10^ m)

Property (unit)	*φ*_00_	*φ*_A_	*φ*_B_	$\varphi_{A}^{-}$	$\varphi_{A}^{+}$
*ε/k*_B_ (K)	11.054	11.063	10.974	11.074	11.052
*r*_m_ (bohr)	5.6039	5.6034	5.6097	5.6034	5.6034
*r*_m_ (Å)	2.9654	2.9652	2.9685	2.9625	2.9652
*σ* (bohr)	4.9873	4.9870	4.9922	4.9868	4.9873
scattering length (Å)	83.68	82.00	96.91	85.30	78.90
effective range (Å)	7.24	7.24	7.30	7.26	7.22
bound state/*k*_B_ (mK)	1.90	1.98	1.39	1.83	2.51

**Table 4:** Integration step sizes used in a given energy range to locate the *n*th zero of *ψ_ℓ_*(*k, r*)

Integration step size (cm^−1^)	Applicable range (cm^−1^)
0.0001	0.0 ≤ *k* ≤ 0.01
0.001	0.01 ≤ *k* ≤ 0.4
0.01	0.4 ≤ *k* ≤ 10.0
0.05	10.0 ≤ *k* ≤ 100.0
0.1	100.0 ≤ *k*

**Table 5:** Relative uncertainties from interpolating between tabulated temperatures

	Max (1 K to 10 K)	Max (10 K to 10^4^ K)	Average (1 K to 10^4^ K)
Δ*B/B ×* 10^6^	187	95.3	18.0
Δ*η*/*η* × 10^6^	107	3.23	3.01
Δλ/λ × 10^6^	85.5	3.24	3.05
Δ*D*_12_/*D*_12_ × 10^6^	1.95	1.93	0.38
Δ*α*_T_*/α*_T_ × 10^6^	288	6.55	6.43

**Table 6:** Relative uncertainty of thermophysical properties of pure ^4^He and ^3^He propagated from the differences between potentials

2000	Δ*B*/*B* × 10^4^	Δ*η*/*η* × 10^4^	Δ*λ*/*λ* × 10^4^	Δ*D*_12_/*D*_12_ ×10^4^	Δ*α_T_*/*α_T_* × 10^4^
2	80	40	40	56	301
5	89	17	17	15	84
10	125	6.3	6.5	5.8	32
20	559	5.4	5.2	4.5	10
50	91	8	8	6.6	7.5
100	43	7.7	7.7	6.4	4.4
200	29	6.7	6.8	5.8	4.5
300	22	6.1	6.2	5.4	4.1
400	19	5.7	5.8	5.1	7.1
500	17	5.5	5.5	4.9	9.8
1000	14	4.8	4.8	4.6	19.9
2000	11	4.6	4.6	5.6	33

**Table 7:** Deviations of *B*_exp_ from *B*_00_ calculated using *φ*_00_

Authors [reference]	Temp. range	<|*B*_exp_ − *B*_00_|>	Range (*B*_exp_ − *B*_00_)	Reported uncertainties
	(K)	(cm^3^·mol^−1^)	(cm^3^·mol^−1^)	(cm^3^·mol^−1^)
Berry [42]	2.60 to 27.10	0.41	0.14 to 0.83	0.20 to 1.00
Gugan and Michel [43] [corrected for *C*(*T*)]	4.22 to 27.10	0.13	0.04 to 0.20	0.20 to 0.70
Gugan and Michel [43]	4.23 to 27.17	0.15	0.03 to 0.33	0.01 to 0.07
Kemp et al. [44]	27.10 to 172.01	0.06	−0.05 to 0.11	0.13 to 0.16
Gammon [45]	98.15 to 1474.85	0.05	−0.07 to 0.07	0.05 to 0.06
Kell et al. [46]	273.15 to 623.15	0.03	−0.05 to 0.05	0.01 to 0.15
Waxman and Davis [8]	298.15	0.08	0.08	0.01
Matacotta et al. [47]	1.47 to 20.30	0.42	−0.14 to 0.96	0.20 to 1.00

**Table 8:** Relative deviations of *η*_exp_ from *η*_00_ calculated using *φ*_00_

	Temperature range (K)	100× (Δ*η*/*η*_00_)_rms_	Range of 100 × Δ*η*/*η*_00_	Reported uncertainties (%)
Wakeham et al. [50]	298 to 793	0.22	0.12 to 0.32	0.2 to 0.5
Maitland and Smith [51]	80 to 2000	−0.51	−2.27 to −0.51	1.5
Vogel [52]	294.5 to 647.9	0.06	0.02 to 0.12	0.3
Kestin et al. [53]	298 to 973	0.20	0.08 to 0.30	0.1 to 0.3
Clark and Smith [54]	77.5 to 373	0.58	0.18 to 1.21	0.5
Dawe and Smith [55]	293 to 1600	−1.16	−2.43 to 0.45	1.0
Coremans et al. [56]	20.4 to 77.8	4.03	1.77 to 6.33	3.0
Kestin and Wakeham [57]	298 to 473	0.16	0.10 to 0.22	0.3
Johnston and Grilly [58]	79 to 296	−0.96	−1.60 to −0.18	3.0
Kalelkar and Kestin [59]	298 to 1121	−0.31	−2.16 to 0.46	0.5
Becker et al. [49] ^4^He	1.3 to 4.2	5.15	−1.65 to 9.57	5.0
Becker et al. [49] ^3^He	1.3 to 4.2	2.73	−0.13 to 4.37	5.0
Kestin et al. [60]	298 to 778	0.35	0.08 to 0.57	0.1 to 0.3
Guevara et al. [61]	1100 to 2150	−0.90	−3.93 to 0.31	0.65

**Table 9:** Relative deviations of *λ*_exp_ from *λ*_00_

	Temperature range (K)	100 × (Δ*λ*/*λ*_00_)_rms_	Range of 100 × Δ*λ*/*λ*_00_	Reported uncertainties (%)
Wakeham et al. [50]	298.15 to 973.15	0.23	0.07 to 0.36	0.2 to 0.5
Haarman [62]	328.15 to 468.15	0.43	−0.54 to −0.32	0.3
Jody et al. [63]	400 to 2500	2.34	−4.67 to −0.41	2.0 to 4.7
Assael et al. [64]	308.15	0.20	−0.20	0.2
Acton and Kellner [65]	3.3 to 20.0	0.66	−0.34 to −1.30	1.0
Kestin et al. [66]	300.65	0.13	−0.13	0.3

**Table 10:** Deviations of the *D*_12,exp_ from *D*_12,00_ calculated using *φ*_00_

	Temperature range (K)	100 × (Δ*D*/*D*_00_)_rms_	Range of 100 × Δ*D*/*D*_00_	Reported uncertainties (%)
Liner and Weissman [67]	303 to 806	1.613	−4.4 to 0.55	1.3 to 4.7
Bendt [68]	14.4 to 296.0	3.876	−6.9 to 5.6	2.0 to 4.0
DuBro and Weissman [69]	76.5 to 888.3	4.142	−6.7 to −1.1	5.0

## 8. Appendix A. Calculations

The results of the present calculations for ^4^He, ^3^He, and their equimolar mixture are listed in Tables A1, A2, and A3, respectively. These tables contain the second virial coefficient for the pure species, the interaction second virial coefficient *B*_12_, the zero-density viscosity and thermal conductivity, the diffusion coefficient, and the thermal diffusion coefficient. Derivatives with respect to temperature are provided to facilitate interpolation and for use in calculating acoustic virial coefficients. The tables for pure ^4^He and ^3^He contain the self-diffusion coefficient, and the thermal diffusion factor of binary mixtures of ^4^He and ^3^He with mole fractions of 0.99999 and 0.00001, respectively.

**Table A1:** Thermophysical properties of ^4^He as a function of temperature, where (−2) is × 10^−2^

*T*	*B*	d*B*/d*T*	d^2^*B*/d*T*^2^	*η*	d*η*/d*T*	*λ*	d*λ*/d*T*	*D*(101.3 kPa)	*α_T_*
(K)	(cm^3^·mol^−1^)	(cm^3^·mol^−1^·K^−1^)	(cm^3^·mol^−1^·K^−2^)	(*μ* Pa·s)	(*μ* Pa·K^−1^)	(mW·m^−1^·K^−1^)	(mW·m^−1^·K^−2^)	(10^−4^·m^2^·s^−1^)	
1.0	−474.449	664.861	−1771.941	3.279(−1)	5.296(−2)	2.624	4.512(−1)	7.154(−5)	4.147(−2)
1.2	−369.743	411.102	−891.823	3.395(−1)	6.980(−2)	2.713	5.030(−1)	9.622(−5)	5.098(−2)
1.4	−302.255	276.706	−500.007	3.573(−1)	1.096(−1)	2.839	7.759(−1)	1.240(−4)	5.716(−2)
1.6	−255.395	198.357	−304.265	3.834(−1)	1.504(−1)	3.026	1.097	1.560(−4)	6.162(−2)
1.8	−220.996	149.180	−197.490	4.171(−1)	1.860(−1)	3.276	1.395	1.927(−4)	6.501(−2)
2.0	−194.640	116.448	−135.022	4.573(−1)	2.149(−1)	3.581	1.642	2.345(−4)	6.764(−2)
2.25	−169.200	88.972	−89.028	5.146(−1)	2.419(−1)	4.022	1.872	2.943(−4)	7.011(−2)
2.5	−149.421	70.388	−61.860	5.775(−1)	2.600(−1)	4.510	2.023	3.624(−4)	7.190(−2)
2.75	−133.560	57.209	−44.818	6.440(−1)	2.705(−1)	5.028	2.108	4.386(−4)	7.318(−2)
3.0	−120.530	47.499	−33.586	7.123(−1)	2.749(−1)	5.560	2.143	5.228(−4)	7.409(−2)
3.5	−100.334	34.377	−20.391	8.492(−1)	2.708(−1)	6.628	2.110	7.138(−4)	7.525(−2)
4.0	−85.360	26.103	−13.372	9.815(−1)	2.573(−1)	7.658	2.005	9.328(−4)	7.594(−2)
4.5	−73.788	20.526	−9.272	1.106	2.407(−1)	8.630	1.878	1.178(−3)	7.646(−2)
5	−64.566	16.578	−6.706	1.222	2.244(−1)	9.537	1.754	1.446(−3)	7.693(−2)
6	−50.774	11.477	−3.849	1.433	1.974(−1)	11.180	1.547	2.050(−3)	7.784(−2)
7	−40.944	8.419	−2.415	1.620	1.783(−1)	12.650	1.400	2.735(−3)	7.871(−2)
8	−33.581	6.440	−1.615	1.791	1.649(−1)	14.000	1.295	3.495(−3)	7.949(−2)
9	−27.859	5.084	−1.134	1.951	1.551(−1)	15.250	1.218	4.326(−3)	8.014(−2)
10	−23.285	4.114	−8.269(−1)	2.102	1.474(−1)	16.440	1.157	5.226(−3)	8.068(−2)
11	−19.547	3.397	−6.215(−1)	2.246	1.412(−1)	17.570	1.108	6.190(−3)	8.110(−2)
12	−16.435	2.850	−4.790(−1)	2.385	1.359(−1)	18.660	1.066	7.217(−3)	8.143(−2)
14	−11.556	2.087	−3.021(−1)	2.648	1.274(−1)	20.720	9.990(−1)	9.451(−3)	8.186(−2)
16	−7.910	1.591	−2.026(−1)	2.895	1.206(−1)	22.660	9.458(−1)	1.191(−2)	8.208(−2)
18	−5.088	1.251	−1.425(−1)	3.131	1.151(−1)	24.510	9.021(−1)	1.459(−2)	8.216(−2)
20	−2.842	1.007	−1.039(−1)	3.356	1.104(−1)	26.270	8.654(−1)	1.749(−2)	8.214(−2)
22	−1.017	8.27(−1)	−7.81(−2)	3.573	1.064(−1)	27.970	8.338(−1)	2.058(−2)	8.206(−2)
23	−0.227	7.54(−1)	−6.84(−2)	3.678	1.046(−1)	28.800	8.196(−1)	2.220(−2)	8.200(−2)
24	0.494	6.90(−1)	−6.02(−2)	3.782	1.029(−1)	29.610	8.063(−1)	2.387(−2)	8.193(−2)
25	1.155	6.33(−1)	−5.32(−2)	3.884	1.013(−1)	30.410	7.938(−1)	2.558(−2)	8.185(−2)
26	1.762	5.83(−1)	−4.73(−2)	3.985	9.980(−2)	31.200	7.821(−1)	2.734(−2)	8.177(−2)
28	2.840	4.98(−1)	−3.78(−2)	4.181	9.706(−2)	32.740	7.606(−1)	3.101(−2)	8.159(−2)
30	3.766	4.30(−1)	−3.07(−2)	4.373	9.460(−2)	34.240	7.412(−1)	3.485(−2)	8.140(−2)
35	5.587	3.08(−1)	−1.93(−2)	4.833	8.941(−2)	37.840	7.004(−1)	4.522(−2)	8.087(−2)
40	6.917	2.29(−1)	−1.28(−2)	5.269	8.523(−2)	41.260	6.675(−1)	5.664(−2)	8.034(−2)
45	7.921	1.76(−1)	−8.94(−3)	5.686	8.176(−2)	44.530	6.402(−1)	6.907(−2)	7.980(−2)
50	8.698	1.38(−1)	−6.46(−3)	6.087	7.882(−2)	47.670	6.171(−1)	8.246(−2)	7.928(−2)
60	9.806	8.86(−2)	−3.66(−3)	6.851	7.406(−2)	53.650	5.798(−1)	1.120(−1)	7.831(−2)
70	10.537	5.98(−2)	−2.25(−3)	7.572	7.035(−2)	59.290	5.507(−1)	1.452(−1)	7.741(−2)
80	11.038	4.16(−2)	−1.47(−3)	8.260	6.734(−2)	64.680	5.270(−1)	1.818(−1)	7.659(−2)
90	11.389	2.95(−2)	−9.98(−4)	8.920	6.483(−2)	69.846	5.073(−1)	2.216(−1)	7.584(−2)
100	11.640	2.11(−2)	−7.03(−4)	9.558	6.270(−2)	74.833	4.906(−1)	2.646(−1)	7.514(−2)
120	11.947	1.07(−2)	−3.78(−4)	10.775	5.923(−2)	84.360	4.633(−1)	3.597(−1)	7.390(−2)
140	12.098	4.92(−3)	−2.18(−4)	11.932	5.650(−2)	93.405	4.419(−1)	4.666(−1)	7.281(−2)
160	12.160	1.50(−3)	−1.32(−4)	13.039	5.428(−2)	102.063	4.245(−1)	5.847(−1)	7.184(−2)
180	12.167	−6.18(−4)	−8.32(−5)	14.105	5.242(−2)	110.403	4.099(−1)	7.137(−1)	7.097(−2)
200	12.140	−1.96(−3)	−5.34(−5)	15.137	5.083(−2)	118.474	3.975(−1)	8.532(−1)	7.017(−2)
225	12.077	−2.99(−3)	−3.10(−5)	16.386	4.914(−2)	128.241	3.842(−1)	1.042	6.927(−2)
250	11.994	−3.59(−3)	−1.78(−5)	17.596	4.769(−2)	137.701	3.729(−1)	1.246	6.845(−2)
275	11.900	−3.93(−3)	−9.74(−6)	18.772	4.644(−2)	146.897	3.630(−1)	1.466	6.769(−2)
300	11.799	−4.10(−3)	−4.69(−6)	19.919	4.534(−2)	155.863	3.544(−1)	1.700	6.699(−2)
325	11.695	−4.18(−3)	−1.46(−6)	21.040	4.436(−2)	164.625	3.467(−1)	1.949	6.634(−2)
350	11.591	−4.19(−3)	6.15(−7)	22.138	4.348(−2)	173.205	3.398(−1)	2.212	6.573(−2)
375	11.486	−4.15(−3)	1.95(−6)	23.215	4.268(−2)	181.621	3.336(−1)	2.489	6.516(−2)
400	11.383	−4.09(−3)	2.81(−6)	24.272	4.196(−2)	189.889	3.279(−1)	2.780	6.462(−2)
450	11.183	−3.93(−3)	3.66(−6)	26.338	4.069(−2)	206.028	3.179(−1)	3.403	6.361(−2)
500	10.991	−3.74(−3)	3.88(−6)	28.344	3.960(−2)	221.707	3.094(−1)	4.078	6.270(−2)
600	10.637	−3.36(−3)	3.66(−6)	32.211	3.783(−2)	251.926	2.955(−1)	5.584	6.108(−2)
700	10.319	−3.01(−3)	3.19(−6)	35.922	3.643(−2)	280.913	2.846(−1)	7.290	5.968(−2)
800	10.033	−2.72(−3)	2.72(−6)	39.506	3.529(−2)	308.912	2.757(−1)	9.189	5.843(−2)
900	9.774	−2.47(−3)	2.32(−6)	42.986	3.434(−2)	336.095	2.682(−1)	11.278	5.731(−2)
1000	9.538	−2.25(−3)	1.99(−6)	46.378	3.353(−2)	362.590	2.618(−1)	13.551	5.628(−2)
1200	9.124	−1.91(−3)	1.48(−6)	52.946	3.221(−2)	413.878	2.515(−1)	18.639	5.445(−2)
1400	8.770	−1.65(−3)	1.14(−6)	59.280	3.117(−2)	463.334	2.434(−1)	24.430	5.286(−2)
1600	8.462	−1.44(−3)	9.01(−7)	65.427	3.033(−2)	511.329	2.368(−1)	30.909	5.144(−2)
1800	8.190	−1.28(−3)	7.27(−7)	71.421	2.963(−2)	558.122	2.313(−1)	38.060	5.015(−2)
2000	7.947	−1.15(−3)	5.97(−7)	77.286	2.904(−2)	603.905	2.267(−1)	45.872	4.897(−2)
2500	7.437	−9.08(−4)	3.90(−7)	91.499	2.904(−2)	714.835	2.267(−1)	68.240	4.637(−2)
3000	7.026	−7.45(−4)	2.73(−7)	105.216	2.904(−2)	821.876	2.267(−1)	94.577	4.415(−2)
3500	6.684	−6.28(−4)	2.00(−7)	118.560	2.904(−2)	925.992	2.267(−1)	124.803	4.219(−2)
4000	6.393	−5.41(−4)	1.53(−7)	131.612	2.904(−2)	1027.819	2.267(−1)	158.862	4.043(−2)
4500	6.141	−4.73(−4)	1.20(−7)	144.429	2.904(−2)	1127.805	2.267(−1)	196.713	3.882(−2)
5000	5.918	−4.19(−4)	9.68(−8)	157.054	2.904(−2)	1226.279	2.267(−1)	238.324	3.734(−2)
6000	5.542	−3.39(−4)	6.62(−8)	181.847	2.904(−2)	1419.639	2.267(−1)	332.735	3.466(−2)
7000	5.232	−2.83(−4)	4.79(−8)	206.173	2.904(−2)	1609.339	2.267(−1)	441.969	3.229(−2)
8000	4.972	−2.41(−4)	3.61(−8)	230.154	2.904(−2)	1796.322	2.267(−1)	565.960	3.014(−2)
9000	4.747	−2.09(−4)	2.81(−8)	253.873	2.904(−2)	1981.249	2.267(−1)	704.683	2.816(−2)
10000	4.551	−1.84(−4)	2.24(−8)	277.393	2.904(−2)	2164.607	2.267(−1)	858.143	2.633(−2)

**Table A2:** Thermophysical properties of ^3^He as a function of temperature, where (−2) is × 10^−2^

*T*	*B*	d*B*/d*T*	d^2^*B*/d*T*^2^	*η*	d*η*/d*T*	*λ*	d*λ*/d*T*	*D*(101.3 kPa)	*α_T_*
(K)	(cm^3^·mol^−1^)	(cm^3^·mol^−1^·K^−1^)	(cm^3^·mol^−1^·K^−1^)	(*μ* Pa·s)	(*μ* Pa·K^−1^)	(mW·m^−1^·K^−1^)	(mW·m^−1^·K^−2^)	(10^−4^·m^2^·s^−1^)	
1.0	−237.503	174.583	−218.592	5.561(−1)	5.280(−1)	5.756	5.459	1.910(−4)	6.797(−2)
1.2	−206.501	137.501	−156.563	6.608(−1)	5.126(−1)	6.839	5.317	2.706(−4)	8.532(−2)
1.4	−181.829	110.586	−115.265	7.593(−1)	4.688(−1)	7.864	4.893	3.629(−4)	9.794(−2)
1.6	−161.811	90.544	−86.881	8.472(−1)	4.086(−1)	8.786	4.309	4.654(−4)	1.077(−1)
1.8	−145.295	75.286	−66.837	9.224(−1)	3.439(−1)	9.585	3.677	5.756(−4)	1.150(−1)
2.0	−131.470	63.444	−52.348	9.850(−1)	2.830(−1)	10.259	3.073	6.913(−4)	1.200(−1)
2.25	−117.097	52.073	−39.409	1.047	2.187(−1)	10.942	2.421	8.412(−4)	1.235(−1)
2.5	−105.208	43.422	−30.305	1.096	1.701(−1)	11.481	1.909	9.952(−4)	1.247(−1)
2.75	−95.226	36.710	−23.736	1.134	1.360(−1)	11.909	1.535	1.152(−3)	1.242(−1)
3.0	−86.736	31.410	−18.897	1.165	1.136(−1)	12.258	1.275	1.312(−3)	1.224(−1)
3.5	−73.089	23.709	−12.431	1.215	9.298(−2)	12.816	1.004	1.639(−3)	1.171(−1)
4.0	−62.616	18.504	−8.637	1.261	9.003(−2)	13.294	9.302(−1)	1.980(−3)	1.112(−1)
4.5	−54.332	14.836	−6.209	1.306	9.430(−2)	13.762	9.487(−1)	2.338(−3)	1.057(−1)
5	−47.616	12.159	−4.605	1.355	1.006(−1)	14.249	1.002	2.715(−3)	1.012(−1)
6	−37.392	8.599	−2.732	1.461	1.114(−1)	15.310	1.116	3.531(−3)	9.498(−2)
7	−29.972	6.405	−1.752	1.576	1.172(−1)	16.466	1.187	4.431(−3)	9.149(−2)
8	−24.336	4.958	−1.191	1.695	1.191(−1)	17.671	1.217	5.413(−3)	8.964(−2)
9	−19.909	3.953	−8.465(−1)	1.814	1.185(−1)	18.890	1.218	6.475(−3)	8.868(−2)
10	−16.338	3.226	−6.238(−1)	1.931	1.166(−1)	20.102	1.204	7.612(−3)	8.819(−2)
11	−13.397	2.682	−4.732(−1)	2.047	1.141(−1)	21.295	1.181	8.822(−3)	8.795(−2)
12	−10.933	2.264	−3.676(−1)	2.159	1.115(−1)	22.463	1.155	1.010(−2)	8.782(−2)
14	−7.038	1.675	−2.350(−1)	2.377	1.062(−1)	24.720	1.102	1.287(−2)	8.769(−2)
16	−4.101	1.287	−1.593(−1)	2.584	1.014(−1)	26.876	1.054	1.589(−2)	8.757(−2)
18	−1.811	1.018	−1.130(−1)	2.783	9.726(−2)	28.939	1.011	1.916(−2)	8.743(−2)
20	0.022	8.242(−1)	−8.309(−2)	2.974	9.365(−2)	30.923	9.735(−1)	2.266(−2)	8.724(−2)
22	1.519	6.795(−1)	−6.286(−2)	3.158	9.049(−2)	32.837	9.407(−1)	2.639(−2)	8.702(−2)
23	2.168	6.206(−1)	−5.517(−2)	3.248	8.906(−2)	33.770	9.258(−1)	2.834(−2)	8.690(−2)
24	2.762	5.688(−1)	−4.870(−2)	3.336	8.771(−2)	34.688	9.117(−1)	3.035(−2)	8.678(−2)
25	3.308	5.229(−1)	−4.318(−2)	3.423	8.643(−2)	35.593	8.985(−1)	3.240(−2)	8.665(−2)
26	3.810	4.822(−1)	−3.847(−2)	3.509	8.523(−2)	36.486	8.859(−1)	3.451(−2)	8.652(−2)
28	4.703	4.132(−1)	−3.091(−2)	3.677	8.301(−2)	38.234	8.628(−1)	3.889(−2)	8.626(−2)
30	5.471	3.573(−1)	−2.520(−2)	3.841	8.101(−2)	39.939	8.420(−1)	4.348(−2)	8.599(−2)
35	6.987	2.568(−1)	−1.593(−2)	4.235	7.674(−2)	44.033	7.976(−1)	5.580(−2)	8.531(−2)
40	8.097	1.915(−1)	−1.067(−2)	4.610	7.328(−2)	47.928	7.615(−1)	6.933(−2)	8.464(−2)
45	8.936	1.467(−1)	−7.476(−3)	4.969	7.038(−2)	51.658	7.313(−1)	8.400(−2)	8.400(−2)
50	9.586	1.148(−1)	−5.423(−3)	5.314	6.792(−2)	55.249	7.057(−1)	9.978(−2)	8.339(−2)
60	10.509	7.365(−2)	−3.091(−3)	5.973	6.392(−2)	62.088	6.640(−1)	1.345(−1)	8.226(−2)
70	11.114	4.929(−2)	−1.905(−3)	6.596	6.078(−2)	68.559	6.313(−1)	1.734(−1)	8.125(−2)
80	11.524	3.384(−2)	−1.243(−3)	7.190	5.823(−2)	74.735	6.047(−1)	2.161(−1)	8.033(−2)
90	11.808	2.355(−2)	−8.470(−4)	7.762	5.609(−2)	80.667	5.825(−1)	2.627(−1)	7.949(−2)
100	12.005	1.642(−2)	−5.961(−4)	8.313	5.427(−2)	86.394	5.635(−1)	3.128(−1)	7.872(−2)
120	12.237	7.620(−3)	−3.186(−4)	9.367	5.130(−2)	97.341	5.326(−1)	4.236(−1)	7.735(−2)
140	12.336	2.752(−3)	−1.826(−4)	10.369	4.896(−2)	107.740	5.082(−1)	5.479(−1)	7.616(−2)
160	12.360	−1.030(−4)	−1.096(−4)	11.328	4.705(−2)	117.698	4.883(−1)	6.852(−1)	7.510(−2)
180	12.339	−1.842(−3)	−6.771(−5)	12.253	4.545(−2)	127.293	4.717(−1)	8.349(−1)	7.415(−2)
200	12.291	−2.924(−3)	−4.241(−5)	13.148	4.408(−2)	136.581	4.574(−1)	9.968(−1)	7.329(−2)
225	12.206	−3.728(−3)	−2.354(−5)	14.231	4.262(−2)	147.821	4.422(−1)	1.216	7.231(−2)
250	12.107	−4.168(−3)	−1.253(−5)	15.280	4.137(−2)	158.711	4.293(−1)	1.452	7.142(−2)
275	12.000	−4.392(−3)	−5.884(−6)	16.301	4.029(−2)	169.298	4.180(−1)	1.707	7.060(−2)
300	11.889	−4.485(−3)	−1.787(−6)	17.296	3.933(−2)	179.621	4.081(−1)	1.978	6.985(−2)
325	11.776	−4.495(−3)	7.679(−7)	18.268	3.848(−2)	189.710	3.992(−1)	2.266	6.915(−2)
350	11.664	−4.455(−3)	2.364(−6)	19.220	3.772(−2)	199.590	3.913(−1)	2.571	6.849(−2)
375	11.554	−4.383(−3)	3.350(−6)	20.155	3.703(−2)	209.283	3.842(−1)	2.891	6.787(−2)
400	11.445	−4.291(−3)	3.939(−6)	21.073	3.641(−2)	218.804	3.777(−1)	3.228	6.729(−2)
450	11.236	−4.080(−3)	4.413(−6)	22.865	3.531(−2)	237.392	3.662(−1)	3.948	6.621(−2)
500	11.038	−3.857(−3)	4.428(−6)	24.606	3.437(−2)	255.451	3.564(−1)	4.729	6.524(−2)
600	10.673	−3.434(−3)	3.961(−6)	27.962	3.283(−2)	290.258	3.404(−1)	6.469	6.351(−2)
700	10.349	−3.068(−3)	3.370(−6)	31.183	3.162(−2)	323.649	3.278(−1)	8.439	6.201(−2)
800	10.058	−2.758(−3)	2.842(−6)	34.294	3.063(−2)	355.903	3.176(−1)	10.633	6.068(−2)
900	9.796	−2.496(−3)	2.400(−6)	37.314	2.981(−2)	387.217	3.090(−1)	13.044	5.948(−2)
1000	9.557	−2.275(−3)	2.042(−6)	40.259	2.910(−2)	417.739	3.016(−1)	15.669	5.839(−2)
1200	9.139	−1.923(−3)	1.515(−6)	45.959	2.796(−2)	476.824	2.897(−1)	21.541	5.645(−2)
1400	8.782	−1.658(−3)	1.160(−6)	51.457	2.706(−2)	533.800	2.804(−1)	28.225	5.476(−2)
1600	8.472	−1.452(−3)	9.133(−7)	56.793	2.633(−2)	589.092	2.728(−1)	35.699	5.325(−2)
1800	8.199	−1.288(−3)	7.355(−7)	61.996	2.572(−2)	643.000	2.665(−1)	43.949	5.189(−2)
2000	7.955	−1.155(−3)	6.031(−7)	67.087	2.521(−2)	695.745	2.611(−1)	52.961	5.064(−2)
2500	7.443	−9.112(−4)	3.926(−7)	79.424	2.521(−2)	823.544	2.611(−1)	78.760	4.790(−2)
3000	7.031	−7.471(−4)	2.741(−7)	91.331	2.521(−2)	946.864	2.611(−1)	109.131	4.556(−2)
3500	6.688	−6.296(−4)	2.013(−7)	102.913	2.521(−2)	1066.813	2.611(−1)	143.985	4.349(−2)
4000	6.396	−5.417(−4)	1.535(−7)	114.243	2.521(−2)	1184.125	2.611(−1)	183.256	4.164(−2)
4500	6.143	−4.737(−4)	1.206(−7)	125.369	2.521(−2)	1299.316	2.611(−1)	226.896	3.996(−2)
5000	5.920	−4.196(−4)	9.704(−8)	136.328	2.521(−2)	1412.766	2.611(−1)	274.868	3.841(−2)
6000	5.543	−3.393(−4)	6.638(−8)	157.849	2.521(−2)	1635.532	2.611(−1)	383.707	3.561(−2)
7000	5.234	−2.829(−4)	4.799(−8)	178.965	2.521(−2)	1854.081	2.611(−1)	509.625	3.314(−2)
8000	4.973	−2.412(−4)	3.616(−8)	199.781	2.521(−2)	2069.499	2.611(−1)	652.548	3.090(−2)
9000	4.748	−2.093(−4)	2.813(−8)	220.370	2.521(−2)	2282.550	2.611(−1)	812.447	2.885(−2)
10000	4.552	−1.842(−4)	2.243(−8)	240.786	2.521(−2)	2493.792	2.611(−1)	989.327	2.695(−2)

**Table A3:** Thermophysical properties of an equimolar binary mixture of ^3^He ^4^He as a function of temperature, where (−2) is × 10^−2^

*T*	*B*	*dB/dT*	d^2^*B*/d*T*^2^	*η*	d*η*/d*T*	*λ*	*dλ/dT*	*D*(101.3 kPa)	*α_T_*
(K)	(cm^3^·mol^−1^)	(cm^3^·mol^−1^·K^−1^)	(cm^3^·mol^−1^·K^−2^)	(*μ*Pa·s)	(*μ*Pa·K^−1^)	(mW·m^−1^·K^−1^)	(mW·m^−1^·K^−2^)	(10^−4^·m^2^·s^−1^)	
1.0	−338.460	362.783	−761.653	4.147(−1)	2.503(−1)	3.802	2.449	1.152(−4)	5.168(−2)
1.2	−278.477	248.027	−428.348	4.629(−1)	2.363(−1)	4.260	2.185	1.616(−4)	6.401(−2)
1.4	−236.166	180.498	−264.495	5.102(−1)	2.378(−1)	4.690	2.129	2.143(−4)	7.237(−2)
1.6	−204.675	137.383	−174.857	5.582(−1)	2.416(−1)	5.117	2.149	2.732(−4)	7.859(−2)
1.8	−180.297	108.166	−121.639	6.067(−1)	2.437(−1)	5.550	2.175	3.379(−4)	8.327(−2)
2.0	−160.849	87.440	−88.058	6.555(−1)	2.433(−1)	5.986	2.183	4.081(−4)	8.672(−2)
2.25	−141.430	69.017	−61.471	7.159(−1)	2.397(−1)	6.529	2.160	5.032(−4)	8.962(−2)
2.5	−125.902	55.906	−44.632	7.751(−1)	2.338(−1)	7.063	2.108	6.063(−4)	9.129(−2)
2.75	−113.192	46.238	−33.437	8.327(−1)	2.267(−1)	7.582	2.039	7.168(−4)	9.206(−2)
3.0	−102.590	38.900	−25.720	8.884(−1)	2.191(−1)	8.082	1.961	8.346(−4)	9.219(−2)
3.5	−85.895	28.672	−16.178	9.943(−1)	2.046(−1)	9.024	1.810	1.091(−3)	9.134(−2)
4.0	−73.329	22.034	−10.848	1.093	1.921(−1)	9.896	1.685	1.372(−3)	8.988(−2)
4.5	−63.518	17.477	−7.632	1.187	1.820(−1)	10.714	1.589	1.679(−3)	8.838(−2)
5	−55.638	14.211	−5.579	1.276	1.737(−1)	11.490	1.517	2.008(−3)	8.708(−2)
6	−43.756	9.939	−3.248	1.443	1.612(−1)	12.952	1.415	2.734(−3)	8.531(−2)
7	−35.212	7.347	−2.058	1.599	1.519(−1)	14.328	1.341	3.542(−3)	8.444(−2)
8	−28.767	5.654	−1.387	1.747	1.446(−1)	15.639	1.282	4.428(−3)	8.412(−2)
9	−23.731	4.486	−9.800(−1)	1.889	1.384(−1)	16.894	1.230	5.389(−3)	8.407(−2)
10	−19.687	3.646	−7.184(−1)	2.024	1.331(−1)	18.101	1.185	6.420(−3)	8.415(−2)
11	−16.369	3.021	−5.425(−1)	2.155	1.284(−1)	19.265	1.144	7.520(−3)	8.427(−2)
12	−13.597	2.543	−4.199(−1)	2.281	1.242(−1)	20.391	1.108	8.686(−3)	8.439(−2)
14	−9.233	1.872	−2.667(−1)	2.522	1.171(−1)	22.541	1.045	1.121(−2)	8.456(−2)
16	−5.957	1.433	−1.799(−1)	2.751	1.113(−1)	24.578	9.932(−1)	1.397(−2)	8.462(−2)
18	−3.411	1.130	−1.270(−1)	2.968	1.064(−1)	26.519	9.498(−1)	1.697(−2)	8.458(−2)
20	−1.380	9.125(− 1)	−9.303(−2)	3.177	1.022(−1)	28.381	9.127(−1)	2.019(−2)	8.448(−2)
22	0.276	7.508(−1)	−7.015(−2)	3.377	9.863(−2)	30.174	8.807(−1)	2.362(−2)	8.433(−2)
23	0.993	6.851(−1)	−6.148(−2)	3.475	9.701(−2)	31.047	8.663(−1)	2.542(−2)	8.425(−2)
24	1.649	6.273(−1)	−5.418(−2)	3.571	9.549(−2)	31.906	8.527(−1)	2.726(−2)	8.415(−2)
25	2.250	5.763(−1)	−4.799(−2)	3.666	9.406(−2)	32.753	8.399(−1)	2.916(−2)	8.405(−2)
26	2.803	5.311(−1)	−4.271(−2)	3.760	9.271(−2)	33.587	8.279(−1)	3.111(−2)	8.394(−2)
28	3.786	4.545(−1)	−3.424(−2)	3.942	9.024(−2)	35.220	8.058(−1)	3.515(−2)	8.372(−2)
30	4.631	3.927(−1)	−2.786(−2)	4.121	8.800(−2)	36.811	7.858(−1)	3.939(−2)	8.349(−2)
35	6.296	2.818(−1)	−1.754(−2)	4.548	8.328(−2)	40.631	7.436(−1)	5.080(−2)	8.290(−2)
40	7.514	2.100(−1)	−1.172(−2)	4.955	7.945(−2)	44.260	7.094(−1)	6.333(−2)	8.230(−2)
45	8.433	1.609(−1)	−8.188(−3)	5.344	7.627(−2)	47.734	6.810(−1)	7.694(−2)	8.171(−2)
50	9.146	1.260(−1)	−5.929(−3)	5.718	7.357(−2)	51.077	6.569(−1)	9.160(−2)	8.115(−2)
60	10.160	8.104(−2)	−3.370(−3)	6.431	6.919(−2)	57.442	6.177(−1)	1.239(−1)	8.010(−2)
70	10.827	5.450(−2)	−2.074(−3)	7.105	6.576(−2)	63.460	5.871(−1)	1.601(−1)	7.915(−2)
80	11.282	3.769(−2)	−1.353(−3)	7.748	6.297(−2)	69.202	5.622(−1)	1.999(−1)	7.829(−2)
90	11.599	2.650(−2)	−9.213(−4)	8.366	6.065(−2)	74.717	5.414(−1)	2.432(−1)	7.749(−2)
100	11.823	1.874(−2)	−6.493(−4)	8.963	5.866(−2)	80.039	5.237(−1)	2.900(−1)	7.677(−2)
120	12.092	9.153(−3)	−3.476(−4)	10.102	5.544(−2)	90.212	4.948(−1)	3.934(−1)	7.547(−2)
140	12.217	3.832(−3)	−2.001(−4)	11.185	5.290(−2)	99.873	4.722(−1)	5.094(−1)	7.433(−2)
160	12.260	6.941(−4)	−1.209(−4)	12.221	5.083(−2)	109.125	4.537(−1)	6.375(−1)	7.332(−2)
180	12.253	−1.232(−3)	−7.540(−5)	13.220	4.909(−2)	118.039	4.382(−1)	7.774(−1)	7.241(−2)
200	12.215	−2.444(−3)	−4.786(−5)	14.187	4.761(−2)	126.667	4.249(−1)	9.286(−1)	7.159(−2)
225	12.142	−3.361(−3)	−2.725(−5)	15.357	4.603(−2)	137.109	4.108(−1)	1.133	7.065(−2)
250	12.051	−3.879(−3)	−1.515(−5)	16.490	4.468(−2)	147.224	3.988(−1)	1.354	6.980(−2)
275	11.950	−4.159(−3)	−7.805(−6)	17.592	4.351(−2)	157.059	3.883(−1)	1.592	6.901(−2)
300	11.844	−4.294(−3)	−3.233(−6)	18.667	4.248(−2)	166.649	3.791(−1)	1.846	6.829(−2)
325	11.736	−4.336(−3)	−3.446(−7)	19.717	4.156(−2)	176.022	3.709(−1)	2.115	6.762(−2)
350	11.628	−4.320(−3)	1.491(−6)	20.746	4.074(−2)	185.200	3.635(−1)	2.400	6.699(−2)
375	11.520	−4.268(−3)	2.653(−6)	21.755	4.000(−2)	194.204	3.569(−1)	2.700	6.639(−2)
400	11.414	−4.192(−3)	3.375(−6)	22.746	3.932(−2)	203.050	3.509(−1)	3.015	6.583(−2)
450	11.209	−4.004(−3)	4.043(−6)	24.681	3.813(−2)	220.320	3.402(−1)	3.688	6.480(−2)
500	11.014	−3.797(−3)	4.155(−6)	26.562	3.711(−2)	237.099	3.312(−1)	4.419	6.385(−2)
600	10.655	−3.395(−3)	3.811(−6)	30.186	3.545(−2)	269.441	3.163(−1)	6.047	6.219(−2)
700	10.334	−3.040(−3)	3.286(−6)	33.664	3.415(−2)	300.470	3.047(−1)	7.891	6.074(−2)
800	10.045	−2.737(−3)	2.784(−6)	37.023	3.308(−2)	330.444	2.951(−1)	9.943	5.945(−2)
900	9.785	−2.481(−3)	2.361(−6)	40.285	3.219(−2)	359.549	2.872(−1)	12.200	5.830(−2)
1000	9.548	−2.263(−3)	2.014(−6)	43.465	3.143(−2)	387.918	2.804(−1)	14.656	5.724(−2)
1200	9.132	−1.915(−3)	1.499(−6)	49.621	3.019(−2)	442.843	2.693(−1)	20.153	5.536(−2)
1400	8.776	−1.652(−3)	1.150(−6)	55.558	2.922(−2)	495.813	2.607(−1)	26.409	5.373(−2)
1600	8.467	−1.448(−3)	9.072(−7)	61.320	2.843(−2)	547.223	2.537(−1)	33.405	5.227(−2)
1800	8.194	−1.285(−3)	7.313(−7)	66.938	2.778(−2)	597.352	2.478(−1)	41.128	5.094(−2)
2000	7.951	−1.152(−3)	6.002(−7)	72.436	2.722(−2)	646.403	2.428(−1)	49.565	4.973(−2)
2500	7.440	−9.097(−4)	3.913(−7)	85.759	2.613(−2)	765.270	2.332(−1)	73.716	4.707(−2)
3000	7.029	−7.461(−4)	2.734(−7)	98.617	2.534(−2)	879.990	2.261(−1)	102.149	4.479(−2)
3500	6.686	−6.289(−4)	2.008(−7)	111.126	2.472(−2)	991.593	2.206(−1)	134.778	4.279(−2)
4000	6.395	−5.412(−4)	1.532(−7)	123.361	2.424(−2)	1100.756	2.162(−1)	171.543	4.098(−2)
4500	6.142	−4.733(−4)	1.204(−7)	135.376	2.384(−2)	1207.960	2.127(−1)	212.399	3.934(−2)
5000	5.919	−4.193(−4)	9.690(−8)	147.211	2.351(−2)	1313.554	2.098(−1)	257.310	3.783(−2)
6000	5.542	−3.391(−4)	6.630(−8)	170.451	2.300(−2)	1520.927	2.052(−1)	359.205	3.510(−2)
7000	5.233	−2.827(−4)	4.795(−8)	193.255	2.263(−2)	1724.410	2.019(−1)	477.089	3.268(−2)
8000	4.972	−2.411(−4)	3.613(−8)	215.734	2.235(−2)	1925.011	1.994(−1)	610.893	3.049(−2)
9000	4.748	−2.092(−4)	2.811(−8)	237.969	2.213(−2)	2123.436	1.975(−1)	760.589	2.848(−2)
10000	4.551	−1.841(−4)	2.242(−8)	260.016	2.197(−2)	2320.201	1.961(−1)	926.183	2.661(−2)

## References

[1] Guildner LA, Edsinger RE (1976). Deviation of International Practical Temperatures from Thermodynamic Temperatures in the Temperature Range from 273.16 to 730 K. J Res Natl Bur Stand (US).

[2] White MP, Gugan D (1992). Direct Measurements of the Dielectric Virial Coefficients of ^4^He between 3 K and 18 K. Metrologia.

[3] Meyer CW, Reilly ML, Marcarino P (1997).

[4] Moldover MR, Mehl JB, Greenspan J (1986). Gas-filled spherical resonators: Theory and experiment. J Acoust Soc Am.

[5] Gillis KA (1994). Thermodynamic Properties of two Gaseous halogenated Ethers from speed-of-Sound measurements: Difluoromethoxy-Difluoromethane and 2-Difluoromethoxy-1,1,1-Trifluoroethane. Int J Thermophys.

[6] Wilhelm J, Vogel E, Lehmann JK, Wakeham WA (1998). A vibrating-wire viscometer for dilute and dense gases. Int J Thermophys.

[7] Gillis KA, Mehl JB, Moldover MR (1996). Greenspan acoustic viscometer for gases. Rev Sci Instrum.

[8] Waxman M, Davis H (1978). Density of Ultra-Pure Air at 298.15 K for Mass Transfer Buoyancy Corrections. J Res Natl Bur Stand (US).

[9] Tang KT, Toennies JP (1984). An improved simple model for the van der Waals potential based on universal damping functions for the dispersion coefficients. J Chem Phys.

[10] Aziz RA, Slaman MJ (1990). An Analysis of the ITS-90 Relations for the Nonideality of He-3 and He-4− Recommended Relations Based on a New Interatomic Potential for Helium. Metrologia.

[11] Aziz RA, Slaman MJ (1991). An examination of *ab initio* results for the helium potential energy curve. J Chem Phys.

[12] Aziz RA, Janzen AR, Moldover MR (1995). *Ab initio* Calculations for Helium: A standard for Transport Property Measurements. Phys Rev Lett.

[13] Janzen AR, Aziz RA (1997). An accurate potential energy curve for helium based on *ab initio* calculations. J Chem Phys.

[14] Mohr PJ, Taylor BN (1999). The 1998 CODATA Recommended Values of the Fundamental Physical Constants, Web Version 3.1.

[15] Ceperley DM, Partridge H (1986). The He_2_ potential at small distances. J Chem Phys.

[16] Anderson JB, Traynor CA, Boghosian BM (1993). An exact quantum Monte Carlo calculation of the helium-helium intermolecular potential. J Chem Phys.

[17] Klopper W, Noga J (1995). An explicitly correlated coupled cluster calculation of the helium-helium interatomic potential. J Chem Phys.

[18] Korona T, Williams HL, Bukowski R, Jeziorski B, Szalewicz K (1997). Helium dimer potential from symmetry-adapted perturbation theory calculations using large Gaussian geminal and orbital basis sets. J Chem Phys.

[19] Gdanitz RJ (1999). Accurately solving the electronic Schrödinger equation of atoms and molecules using explicitly correlated (*r*_12_)MR-CI IV. The helium dimer (He_2_). Mol Phys.

[20] Van de Bovenkamp J, van Duijneveldt FB (1999). MRCI calculations on the helium dimer employing and interaction optimized basis set. J Chem Phys.

[21] Van Mourik T, Dunning TH (1999). A new *ab initio* potential energy curve for the helium dimer. J Chem Phys.

[22] Bishop DM, Pipin J (1993). Dipole, Quadrupole, Octupole, and Dipole-Octupole Polarizabilities at Real and imaginary Frequencies for H, He, and H_2_ and the Dispersion-Energy Coefficients for Interactions Between Them. Inter J Quant Chem.

[23] Chen M, Chung KT (1996). Retardation long-range potentials between two helium atoms. Phys Rev A.

[24] Komasa J (1999). Exponentially correlated Gaussian functions in variational calculations: Energy expectation values in the ground state helium dimer. J Chem Phys.

[25] Van Mourik T, van Lenthe JH (1995). Benchmark full configuration interaction calculations of the helium dimer. J Chem Phys.

[b26-j55hur] 26R. Gdanitz, private communication.

[27] Bukowski R, Jeziorski B, Szalewicz K (1996). Basis set superposition problem in interaction energy calculations with explicitly correlated bases: Saturated second- and third-order energies for He_2_. J Chem Phys.

[28] Komasa J, Cencek JW, Rychlewski J (1999). Adiabatic corrections of the helium dimer from exponentially correlated Gaussian functions. Chem Phys Lett.

[29] Thakkar AJ (1988). Higher dispersion coefficients: Accurate values for hydrogen atoms and simple estimates for other systems. J Chem Phys.

[30] Jamieson JM, Drake GWF, Dalgarno A (1995). Retarded dipole-dipole dispersion interaction potential for helium. Phys Rev A.

[31] Jansen AR, Aziz RA (1995). Modern He-He Potentials—Another Look at Binding-energy, Effective Range Theory, Retardation, and Efimov States. J Chem Phys.

[32] Hirschfelder JO, Curtiss CF, Bird RB (1964). Molecular Theory of Gases and Liquids.

[33] Hartree DR (1958). Numerical Analysis.

[34] LeRoy RJ University of Waterloo Chemical Physics Report.

[35] Hurly JJ, McConville GT, Taylor WL (1990). Algorithms and Fortran Programs to Calculate Quantum Collision integrals for Realistic Intermolecular Potentials, MLM-3635.

[36] Maitland GC, Rigby M, Smith EB, Wakeham WA (1981). Intermolecular Forces.

[37] Kahaner DK, Moler C, Nash S (1989). Numerical Methods and Software.

[38] Meeks FR, Cleland TJ, Hutchinson KE, Taylor WL (1984). On the quantum cross sections in dilute gases. J Chem Phys.

[39] Viehland LA, Janzen AR, Aziz RA (1995). High approximations to the transport properties of pure atomic gases. J Chem Phys.

[40] Assael MJ, Wakeham WA, Kestin J (1980). Higher-Order Approximation to the Thermal Conductivity of Monatomic Gas Mixtures. Int J Thermophys.

[41] Mason EA (1957). Higher Approximations for the Transport Properties of Binary Gas Mixtures I. General Formulas. J Chem Phys.

[42] Berry KH (1979). NPL-75: A low Temperature Gas Thermometry Scale from 2.6 K to 27.1 K. Metrologia.

[43] Gugan D, Michel GW (1980). Dielectric Constant Gas Thermometry from 4.2 to 27.1 K. Metrologia.

[44] Kemp RC, Kemp WRG, Besley LM (1986). A Determination of Thermodynamic Temperatures and Measurements of the Second Virial Coefficient of ^4^He Between 13.81 K and 287 K Using a Constant-Volume Gas Thermometer. Metrologia.

[45] Gammon BE (1979). The velocity of sound with derived state properties in helium at −175 to 150 °C with pressure to 150 atm. J Chem Phys.

[46] Kell GS, McLaurin GE, Whalley E (1978). Second virial coefficient of helium from 0 to 500 °C by the two-temperature gas-expansion method. J Chem Phys.

[47] Matacotta FC, McConville GT, Steur PPM, Durieux M (1978). Measurements and Calculations of the ^3^He Second Virial Coefficient Between 1.5 K and 20.3 K. Metrologia.

[48] McConville GT, Hurly JJ (1991). An Analysis of the Accuracy of the Calculation of the Second Virial Coefficient of Helium from Interatomic Potential Functions. Metrologia.

[49] Becker EW, Misenta R, Schmeissner F (1954). Die Zähigkeit von gasförmigem He^3^ and He^4^ zwischen 1,3 °K. Z Phys.

[50] Wakeham WA, Nagashima A, Sengers JV (1991). Measurement of the transport properties of fluids.

[51] Maitland GC, Smith EB (1971). Critical Reassessment of Viscosities of 11 Common Gases. J Chem Eng Data.

[52] Vogel E (1984). Präzisionsmessungen des Viskositätskoeffizienten von Stickstoff und den Edelgases zwischen Raumtemperatur und 650 K. Ber Bunsenges Phys Chem.

[53] Kestin J, Ro ST, Wakeham WA (1972). Viscosity of the Noble Gases in the Temperature Range 25-700 °C. J Chem Phys.

[54] Clarke AG, Smith EB (1969). Low-Temperature Viscosities and Intermolecular Forces of Simple Gases. J Chem Phys.

[55] Dawe RA, Smith EB (1970). Viscosities of the Inert Gases at High Temperatures. J Chem Phys.

[56] Coremans JMJ, Van Itterbeek A, Beenakker JJM, Knaap HFP, Zandbergen P (1958). The viscosity of gaseous He, Ne, H_2_, and D_2_ below 80 °K. Physica.

[57] Kestin J, Wakeham WA (1983). The Viscosity and Diffusion Coefficient of Binary Mixtures of Nitrous Oxide with He, Ne and CO. Ber Bunsenges Phys Chem.

[58] Johnston HL, Grilly ER (1942). Viscosities of Carbon Monoxide, Helium, Neon, and Argon between 80° and 300 °K. Coefficients of Viscosity. J Phys Chem.

[59] Kalelkar AS, Kestin J (1970). Viscosity of He-Ar and He-Kr Binary Gaseous Mixtures in the Temperature Range 25-700 °C. J Chem Phys.

[60] Kestin J, Khalifa HE, Wakeham WA (1978). The Viscosity and Diffusion Coefficients of the Binary Mixtures of Xenon with the other Noble Gases. Physica.

[61] Guevara FA, McInteer BB, Wageman WE (1969). High-Temperature Viscosity Ratios for Hydrogen, Helium, Argon, and Nitrogen. Phys Fluids.

[62] Haarman JW (1973). Thermal Conductivity Measurements of He, Ne, Ar, Kr, N_2_, and CO_2_ with a Transient Hot Wire Method. AIP Conf Proc.

[63] Jody BJ, Saxena SC, Nain VPS, Aziz RA (1977). Thermal Conductivity of Helium: A Probe for the Repulsive Wall of the Interatomic Potential. Chem Phys.

[64] Assael MJ, Dix M, Lucas A, Wakeham WA (1981). Absolute Determination of the Thermal Conductivity of the Noble Gases and Two of their Binary Mixtures as a Function of Density. J Chem Soc, Faraday Trans.

[65] Acton A, Kellner K (1977). The Low Temperature Thermal Conductivity of ^4^He. Physica.

[66] Kestin J, Paul R, Clifford AA, Wakeham WA (1980). Absolute Determination of the Thermal Conductivity of the Noble Gases at Room Temperature up to 35 MPa. Physica.

[67] Liner JC, Weissman S (1971). Determination of the Temperature Dependence of Gaseous Diffusion Coefficients Using gas Chromatographic Apparatus. J Chem Phys.

[68] Bendt PJ (1958). Measurements of He^3^-He^4^ and H_2_-D_2_ gas diffusion Coefficients. Phys Rev.

[69] DuBro GA, Weissman S (1970). Measurements of Gaseous Diffusion Coefficients. Phys Fluids.

[70] Moldover MR (1998). Can a pressure standard be based on capacitance measurements?. J Res Natl Inst Stand Technol.

[71] Hurly JJ, Taylor WL, Meeks FR (1992). Thermal-Diffusion Factors at Low-Temperatures for Gas-Phase Mixtures of Isotopic Helium. J Chem Phys.

[72] Taylor WL (1972). Thermal Diffusion factor for the ^3^He-^4^He system in the quantum region. J Chem Phys.

[73] Taylor WL, Weissman S (1971). Thermal Diffusion Factors for the ^3^He-^4^He system. J Chem Phys.

[74] McInteer BB, Aldrich LT, Nier AO (1947). The Thermal Diffusion Constant of helium and the Separation of He^3^ by Thermal Diffusion. Phys Rev.

[75] Watson WW, Howard AJ, Miller NE, Shiffrin RM (1963). Isotopic Thermal Diffusion Factors for Helium and neon at Low Temperatures. Z Naturforsch Teal A.

